# Dual‐Stimuli Responsive Poly(*N*‐acryloyl glycine) Microgels: Investigation of Network Structures and Application for Loading and Release of Biologics

**DOI:** 10.1002/smll.75268

**Published:** 2026-08-20

**Authors:** Yusuf Dursun, Burak Aydin, Bidit Lamsal, Deniz Ceylan, Kay Saalwächter, Christian Wischke

**Affiliations:** ^1^ Institute of Pharmacy Martin‐Luther‐University Halle‐Wittenberg Halle Germany; ^2^ Faculty of Pharmacy Bezmialem Vakıf University Istanbul Turkey; ^3^ Institute of Physics Martin‐Luther‐University Halle‐Wittenberg Halle Germany

**Keywords:** biopharmaceuticals, controlled release, drug encapsulation, hydrogel microparticles, microfluidics, Poly(*N*‐acryloyl glycine), swelling‐mediated loading

## Abstract

Poly(*N*‐acryloyl glycine) (PNAG) bulk hydrogels are known to exhibit pH‐ and ion‐responsive behavior. In this study, PNAG hydrogel particles were prepared by droplet‐based microfluidics in combination with in situ photopolymerization. Rheological investigations led to the optimization of the PNAG gelation kinetics by the choice of photoinitiators, buffer media (pH 3, 7.4, or 9), and calcium ion (Ca^2+^) concentrations (none or 5 wt.% CaCl_2_). The microparticles demonstrated a strong swelling response, with a volume increase of over 5000 vol.% upon pH shifts. Rheology and multiple‐quantum nuclear magnetic resonance (MQ‐NMR) further revealed pronounced differences in network constraints and chain mobility between the investigated conditions. Via pH‐induced swelling, model biopharmaceuticals such as glutathione (GSH) were loaded into the particles by soaking, followed by compound entrapment through Ca^2+^‐induced network constriction, leading to high drug payloads >20 wt.%. Drug release studies revealed an initial burst release, followed by a remarkable prolonged diffusion‐controlled release for about 2 weeks. As PNAG hydrogel particles demonstrated their potential for drug delivery, further studies could explore the tailoring of mesh sizes and broaden the spectrum of investigated biopharmaceuticals.

## Graphical abstract

Poly(*N*‐acryloyl glycine) hydrogel microparticles were fabricated via droplet‐based microfluidics and in situ photopolymerization. Rheology and MQ‐NMR revealed pronounced effects of pH and Ca^2^^+^ on network constraints and chain mobility, governing extensive swelling. Dual‐stimuli‐responsive swelling enabled post‐production loading of biopharmaceuticals at high payloads, followed by prolonged release.

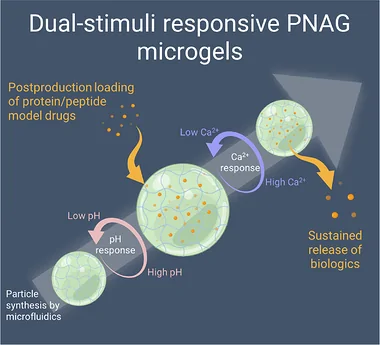

## 1 Introduction

The application of biopharmaceuticals, including proteins and peptides, offers promising therapeutic options for many common diseases such as cancer, diabetes, infections, and inflammatory diseases. These drugs often enable high selectivity, specificity, and efficacy of treatment [1]. However, their structural fragility poses significant challenges for drug delivery, as even minor structural changes during manufacturing, storage, or application can impair therapeutic efficacy or induce immunogenicity [1, 2]. Thus, carrier systems capable of hosting biopharmaceuticals such as peptides and proteins are of particular interest.

Hydrogels can mimic physiological conditions and are, therefore, promising candidate materials for the sustained release of sensitive compounds [3]. More specifically, hydrogels composed of poly(*N*‐acryloyl glycine) (PNAG) have demonstrated a number of interesting features, including responsiveness to pH‐, temperature‐, and multivalent ions. They were also positively evaluated in vivo upon contact with cells and tissues, suggesting good biocompatibility [4, 5, 6]. The stimuli‐responsiveness of PNAG originates from carboxylic groups on its glycine side chains [4]. Below the p*K*_a_ ∼4 (based on the monomer's p*K*_a_ value (3.84) [7]), the carboxylic groups of PNAG are protonated, enabling the formation of intra‐ and intermolecular hydrogen bonds within the entangled physical polymer network. At higher pH values, deprotonation induces ionic repulsion of carboxylate moieties, loosening the network and allowing for more water uptake. Multivalent cations, such as calcium ions (Ca^2+^), can establish ionic crosslinks between carboxylate groups as a secondary type of physical crosslink, as suggested in prior studies [4]. The pH‐ and ion‐dependent interactions are illustrated in Figure 1.

**FIGURE 1:**
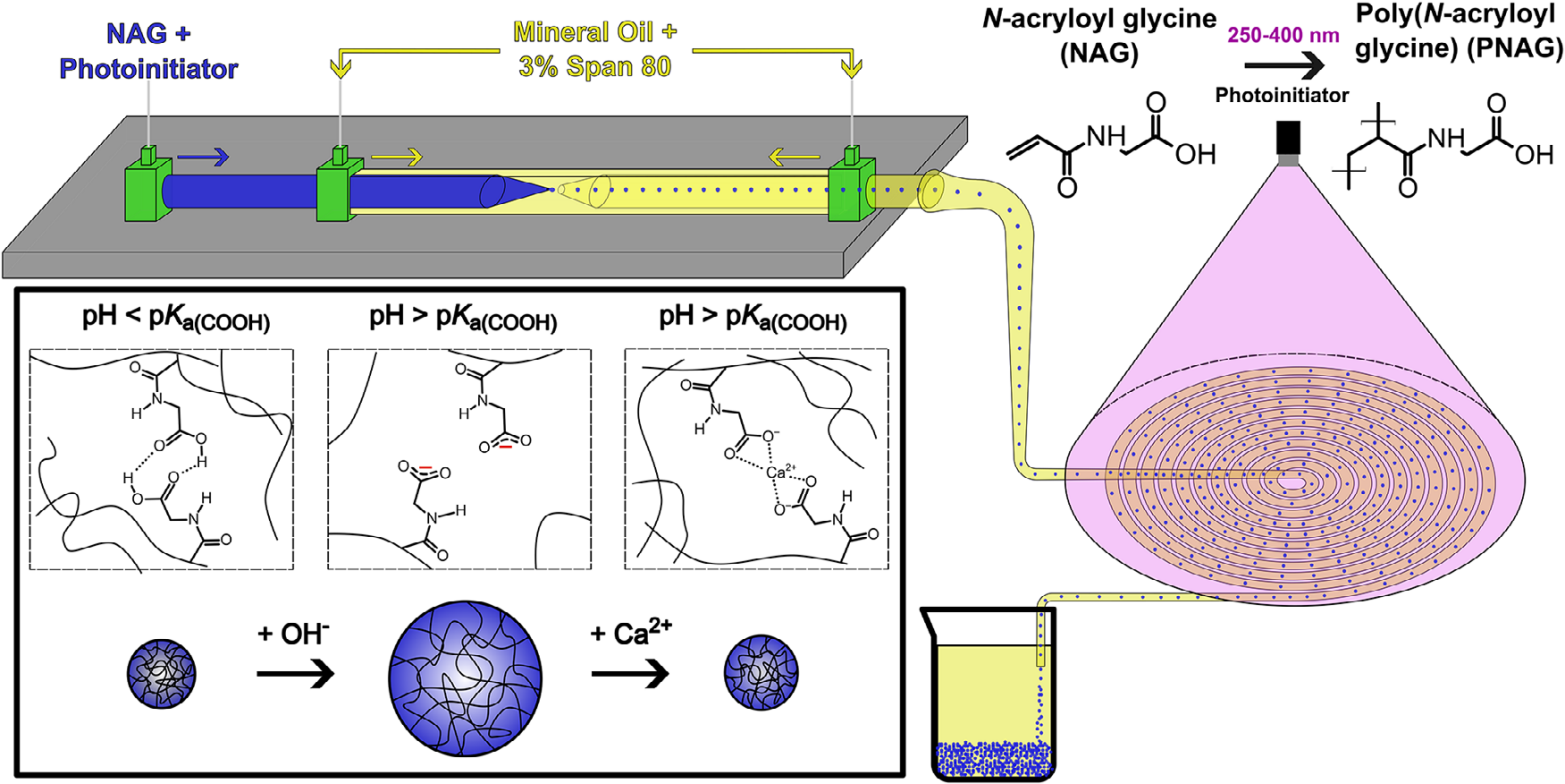
Schematic representation of the synthesis strategy for poly(*N*‐acryloyl glycine) microparticles and the underlying pH‐ and calcium ion‐responsive mechanism.

These non‐covalent interactions are reversible, making PNAG network stability dependent on the conditions that influence their association and dissociation. Literature suggests that bulk gels from PNAG remain stable under acidic conditions and at elevated Ca^2+^ concentrations, whereas they eventually dissolved in buffers at higher pH [4]. Although gels prepared at different monomer concentrations were compared in this study [4], the influence of polymerization parameters on gel formation and stability was not systematically investigated. In another study, the impact of photoinitiator concentration, light intensity, monomer concentration, and polymerization temperature on bulk PNAG gel formation and properties were explored [8], where photoinitiator concentration strongly influenced polymer molecular weight, whereas monomer concentration had a pronounced effect on chain entanglement and gel formation.

To date, PNAG‐based hydrogels have been reported in the form of nanoparticles [5, 9], bulk hydrogels [4, 5, 6, 8], sponges [10], and 3D‐printed scaffolds [11]. Except for bulk hydrogels, these systems typically incorporated PNAG as part of a composite material rather than in its pure form. To our knowledge, PNAG hydrogel microparticles, particularly those with narrow size distributions as relevant for injectable formulations, have not yet been reported. Accordingly, a target particle diameter of approximately 50 µm was selected, lying within the 10–100 µm range reported as relevant for subcutaneous and intramuscular depot administration [12].

Droplet‐based microfluidics was selected for the generation of PNAG microparticles, as this method offers precise control over particle size and uniformity, both of which are critical factors for consistent drug delivery performance [13]. Unlike conventional emulsion‐based methods, which rely on vigorous stirring and can deform partially polymerized particles, microfluidics operates in a controlled environment that minimizes mechanical stress. Additionally, this method enables continuous and uniform UV exposure of particles for a predefined time, which improves bulk homogeneity, reproducibility, and reaction control [14].

Despite the interesting features of PNAG hydrogels as delivery systems for biopharmaceuticals, the synthesis conditions of PNAG are incompatible with peptide/protein stability. PNAG hydrogels are generally prepared by free radical polymerization of the monomer, *N*‐acryloyl glycine (NAG), in acidic aqueous solution using initiators either triggered by UV irradiation [4, 6] or at elevated temperatures [5]. Polymerization outside acidic conditions leads to gel structures that are physically less stable, at least in the absence of multivalent cations. Given the negative effects of PNAG synthesis conditions on the structural stability of biopharmaceuticals [15], there is a demand for alternative concepts of drug loading of PNAG materials.

In this study, a number of peptides/proteins were used as model biopharmaceuticals. Out of these, glutathione (GSH), a tripeptide composed of glutamic acid, cysteine, and glycine, has therapeutic relevance as the body's most powerful antioxidant, with its thiol (─SH) group acting as a potent reducing agent. It plays a critical role in maintaining cellular redox balance and detoxification [16]. GSH offers therapeutic benefits in conditions associated with oxidative stress, such as neurodegenerative diseases [17], viral infections [18], cancer therapy [19], and inflammatory disorders [20]. As a supplement, oral administration of GSH is inefficient due to poor bioavailability, making parenterally administered dosage forms such as microparticles a more viable option [21]. GSH is highly susceptible to environmental stress, undergoing oxidation into glutathione disulfide (GSSG). This sensitivity makes it an appropriate model for assessing the stability of biopharmaceuticals during drug delivery [22], which is also one of the major chemical instability mechanisms affecting proteins.

To address the limitations of traditional PNAG hydrogel synthesis, we propose a drug loading strategy that is applied after synthesizing blank PNAG hydrogel microparticles in microfluidic channels (Figure 1). This loading approach utilizes the responsiveness of the hydrogel to different types of stimuli and provides the possibility of encapsulation under mild conditions. Specifically, particles will be allowed to swell in a drug solution above the polymer's p*K*_a_. Subsequent deswelling will be triggered by the addition of Ca^2+^ for encapsulating the drug within the (constricting) hydrogel network. This swelling‐deswelling mechanism may enable drug loading of different types of biopharmaceuticals. Additionally, facilitating the dual‐stimuli‐sensitivity of PNAG is expected to achieve higher drug loading levels compared to passive loading via pure pore diffusion into already ionically crosslinked hydrogel microparticles.

## 2 Results and Discussion

### 2.1 Identification of Reaction Conditions for PNAG Hydrogel Synthesis

#### 2.1.1 Effect of Photoinitiator on Gelation

For a successful synthesis of PNAG hydrogel microparticles in a microfluidic setup, it is essential to ensure that polymerization is completed within the residence time of particles in the microfluidic device. Incomplete polymerization would compromise particle performance; therefore, it is particularly important to understand and control the kinetics of polymerization‐induced gelation of PNAG. In a systematic approach, model studies of the photopolymerization of thin film NAG samples were conducted using photorheology. These studies aimed to elucidate the effect of key parameters such as photoinitiator type and concentration, and to rationalize the reaction conditions selected for subsequent microfluidic hydrogel particle synthesis.

As shown in Figure 2A, the gelation kinetics were predominantly influenced by the monomer concentration. At high monomer concentration (25 wt.%), gelation occurred within seconds, whereas at low monomer concentration (5 wt.%), the gelation time exceeded 7 min. This suggests that gelation is limited at low monomer concentrations by the availability of monomers to sustain polymer chain propagation. In contrast, at high concentrations, gelation is accelerated by the abundance of reactive sites. Interestingly, photoinitiator concentration had minimal impact on gelation speed at both low and high monomer concentrations.

**FIGURE 2:**
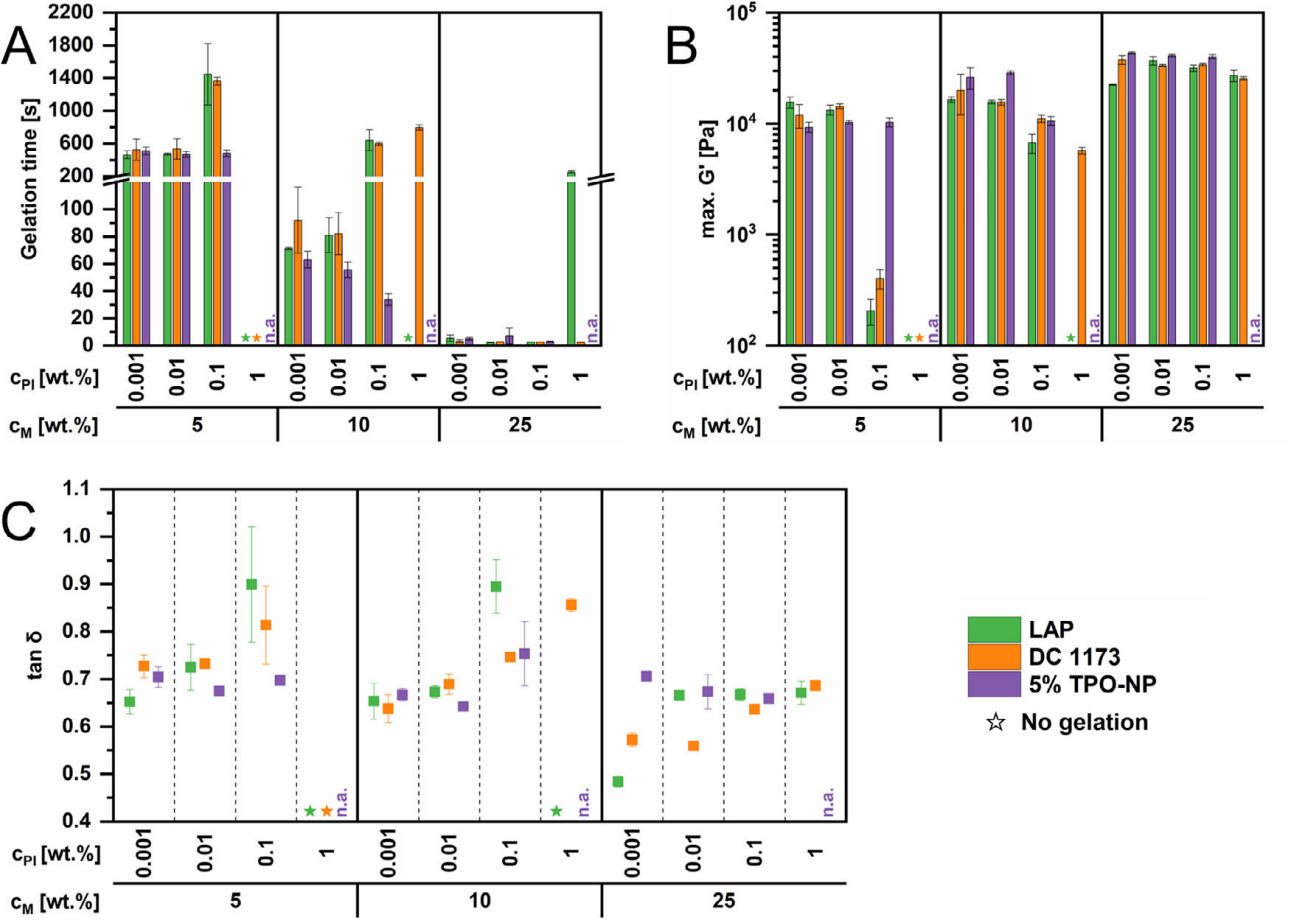
Effect of photoinitiator type and concentration on PNAG gelation as investigated by in situ photopolymerization of thin film samples. NAG monomer concentrations (c_M_) were 5, 10, and 25 wt.%, and photoinitiator concentrations (c_PI_) ranged from 0.001 to 1 wt.%. (A) Gelation time, (B) Maximum storage modulus (G′ max), and (C) Loss factor (tan δ). Conditions marked with a star (★) did not consistently achieve gelation across all repetitive measurements; n.a., not analyzed because of limited photoinitiator solubility. Data are presented as mean ± standard deviation (*n* = 3).

At medium monomer concentration (10 wt.%), the type and concentration of photoinitiator played an important role. TPO‐NP (diphenyl(2,4,6‐trimethylbenzoyl)phosphine oxide nanoparticles) facilitated the fastest gelation; at higher concentrations, such as 0.1%, the gelation time was reduced from 60 s to approx. 30 s. Conversely, increasing concentrations of LAP (lithium phenyl‐2,4,6‐trimethylbenzoylphosphinate) and DC1173 (2‐hydroxy‐2‐methylpropiophenone) photoinitiators delayed the gelation or resulted in incomplete gelation, with samples appearing as a cloudy puddle rather than a gel. This observation is likely attributed to the excessive generation of radicals at high concentrations of LAP and DC1173 that interact with the alkene groups of NAG to initiate free radical polymerization [23]. However, at elevated photoinitiator levels, the excess of radicals also promotes radical‐radical interactions, leading to premature termination of propagating chains. This results in short polymer chains that lack the necessary entanglement required for forming a physical hydrogel network. Even at high monomer concentration, excessive LAP concentrations caused substantial delays in gelation. In contrast, TPO‐NP (which contains only 5% TPO) has one of the highest extinction coefficients among commonly used photoinitiators and is a highly efficient PI [24] despite its lower overall concentration. The strong absorption of TPO may lead to an intense but transient burst of radicals upon UV exposure, initiating many chains early. Because most of the initiator is consumed during this initiation phase, fewer new radicals are generated later on, thereby lowering the chance of early termination events during propagation. Accordingly, TPO‐NP ultimately allowed for a more effective crosslinking, outperforming the other photoinitiators.

The maximum storage modulus (Figure 2B) exhibited trends similar to those observed for gelation kinetics, with monomer concentration being the dominant factor. Higher photoinitiator levels slightly reduced max. G′, potentially due to a decrease in average molecular weight or diminished chain entanglements resulting from faster polymerization kinetics. Among the photoinitiators, gels prepared with TPO‐NP exhibited higher and generally more consistent max. G′ values across replicate samples.

The loss factor (Figure 2C) offers additional insights into gel properties by evaluating the balance between viscous and elastic contributions, with lower values (tan*δ* <<1) indicating profound gel characteristics. The photoinitiator concentration had a larger effect on the loss factor than monomer concentration, especially for LAP and DC1173. At higher photoinitiator levels, tan*δ* increased for these systems, supporting the hypothesis of premature termination of propagating polymer chains. Such less developed physical networks result in gels with a larger viscous component in their rheological behavior. Conversely, TPO‐NP maintained a stable gelation performance. These gels were marginally affected by changes in TPO‐NP concentration.

In summary, TPO‐NP was selected as the most suitable photoinitiator due to its efficiency already at low concentrations and its consistent performance. Overall, considering both the gel stiffness and loss factor, the resulting PNAG gels can be characterized as soft, viscoelastic solids that hold their shape at rest but deform readily under applied pressure.

#### 2.1.2 Effect of pH and Ca^2+^ on Gelation

As a next step, the effects of pH and Ca^2+^ on gelation kinetics and gel properties were investigated by photorheology, using TPO‐NP as the photoinitiator. Aqueous solutions of the monomer NAG naturally exhibit a pH around 3. We hypothesized that performing NAG polymerization at higher pH values would slow gel formation kinetics and reduce the maximum storage modulus due to deprotonation of the carboxyl groups, resulting in increased electrostatic repulsion. This repulsion may impair chain propagation by decreasing monomer accessibility to growing radical chain ends [25]. Additionally, the resulting physical polymer network may be destabilized by electrostatic repulsion between negatively charged chains.

The addition of Ca^2+^ was expected to counteract the charge repulsion at higher pH values by ionic crosslinking, thereby improving the integrity of the entangled polymer network and promoting faster gelation and a higher maximum storage modulus compared to networks stabilized solely by hydrogen bonds. To investigate maximum possible effects, a high CaCl_2_ concentration of 5 wt.% was used, in some cases approaching solubility limits in mixture with the other reagents. Beyond its role in physical stabilization of the gel network, Ca^2+^ may also directly influence the polymerization process by shielding negative charges and thereby reducing electrostatic hindrance to polymer chain propagation [26].

First, gelation time was studied. As shown in Figure 3A, and in accordance with the hypothesis, gelation time increased with rising pH, as exemplified for 10 wt.% monomer concentration (see Figure 3A’), where a clear jump of gel point was observed for all photoinitiator concentrations, when switching from pH 3 to pH 7.4. Under the conditions of the experiment, where the sample volume in the rheometer is fixed and no exchange of medium occurs, this observation indicates that more time is required to form stable network structures that immobilize the aqueous phase. Higher photoinitiator concentrations generally shortened gelation time, particularly at low monomer concentration.

**FIGURE 3:**
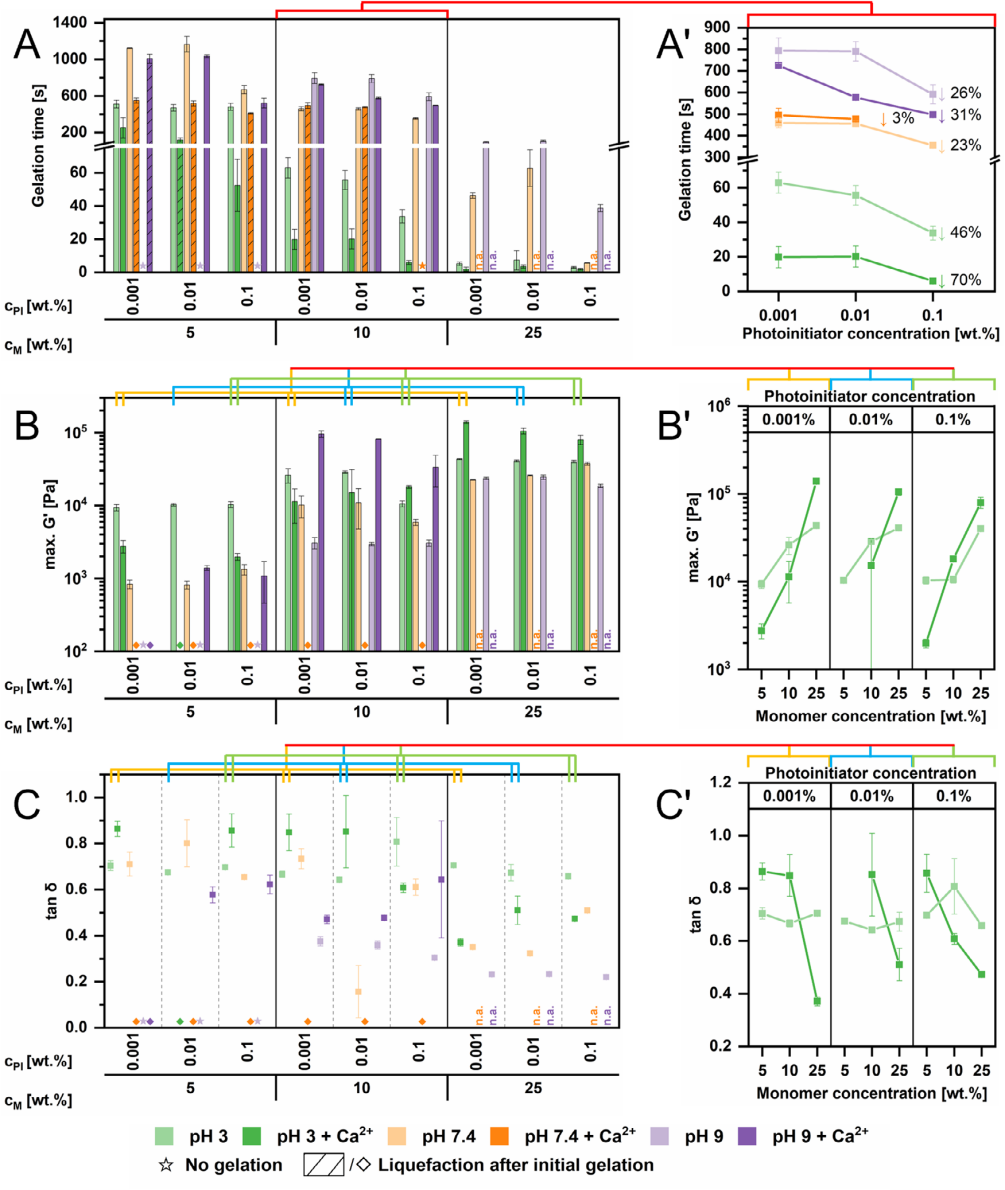
Effect of pH and Ca^2+^ on PNAG gelation. Samples were prepared at pH 3 (green), 7.4 (orange), and 9 (purple), with (darker shades) and without (lighter shades) the presence of Ca^2+^ during photopolymerization. (A, A′) Gelation time, (B, B′) Maximum storage modulus (G′ max), and (C, C′) Loss factor (tan δ). Conditions marked with a star (★) did not consistently achieve gelation across all repetitive measurements; n.a., not analyzed, as precipitation occurred shortly after mixing the components due to exceeding solubility limits. Hatched columns or conditions marked with a diamond (◆) indicate samples that experienced liquefaction after initial gelation. Data are presented as mean ± standard deviation (*n* = 3).

The addition of Ca^2+^ shortened gelation time at pH 7.4 and pH 9, though not to the expected extent. In contrast, under acidic conditions, Ca^2+^ induced a relatively stronger decrease in the gelling time (Figure 3A′). While most ionizable groups should theoretically remain protonated (─COOH) at pH 3, Ca^2+^ still had a considerable effect. This is because Ca^2+^ can interact with the fraction of free carboxylate groups (─COO^−^), e.g., by integration into interchain‐crosslinks via complexation. Additionally, Ca^2+^ may interact with carboxylate moieties in a uni‐ or bidentate mode, stabilizing them in their deprotonated state [27]. Eventually, under certain conditions, initially protonated groups may also release protons and bind Ca^2+^ through electrostatic exchange with the partial charge of oxygen atoms (ion‐dipole interaction), further stabilizing the polymer structure [28, 29]. It should be noted that the use of Ca^2+^ was limited in some conditions: Above the p*K*_a_ of NAG and at high monomer concentrations, a precipitate, likely involving Ca^2+^ and NAG, was observed.

At elevated pH, in some cases, the gels softened back into a solution or experienced a rapid drop in storage modulus after initial gelation (affected samples are marked in Figure 3A as hatched columns and with asterisks). This phenomenon occurred predominantly at near‐neutral pH in the presence of Ca^2+^ and was less common under acidic or basic conditions. At near‐neutral pH, ionic crosslinking via Ca^2+^ leads to junctions between polymer chains being in close proximity, while electrostatic repulsion between other carboxylate groups keeps unbound chains apart. These opposing forces may put the physical network under tension during its formation, making it more prone to mechanical failure during polymerization, eventually leading to liquefaction. In contrast, at basic pH, although the degree of deprotonation is similarly high compared to near‐neutral pH (pH 7.4: ∼99.96% vs pH 9: ∼100%, calculated based on a p*K*_a_ of 4), the greater presence of hydroxide ions (and monovalent counter‐ions like Na^+^) may screen or mitigate electrostatic interactions between polymer chains. This screening allows for greater conformational flexibility of the chains, reducing tension and preventing network failure. This interpretation is supported by data shown in Figure 3A: at near‐neutral pH, gelation occurred faster than under basic conditions, and the intermediate storage modulus reached directly before liquefaction was generally higher (e.g., for c_M_ = 5 wt.% and c_PI_ = 0.01 wt.%: G′ ≈ 1110 Pa at pH 7.4 + Ca^2+^ vs. G′ ≈ 7.8 Pa at pH 9.0 + Ca^2+^).

Second, the mechanical properties of successfully formed gels were tested, showing clear differences depending on synthesis conditions (Figure 3B). With increasing pH, the gels generally became less stiff (e.g., for c_M_ = 10 wt.% and c_PI_ = 0.01 wt.%: G′ ≈ 28.7 ± 1.3 kPa at pH 3 vs. G′ ≈ 3.0 ± 0.1 kPa at pH 9). This reduced stiffness at high pH can be attributed to electrostatic repulsion after deprotonation of carboxyl groups under these conditions, which disrupts network stabilization. A direct comparison of Ca^2+^‐stabilized vs. non‐stabilized gels at elevated pH was only possible at pH 9 for 10 wt.% monomer solutions (e.g., for c_PI_ = 0.01 wt.%: G′ ≈ 3.0 ± 0.1 kPa at pH 9 vs. G′ ≈ 81.2 ± 0.4 kPa at pH 9 + Ca^2+^), as under other conditions either no gelation occurred or Ca^2+^ addition caused liquefaction. While ionic crosslinks are generally stronger than hydrogen bonds [30], this did not universally translate into stiffer Ca^2+^‐containing PNAG gels at high pH; the maximum storage modulus exceeded the values measured for acidic gels only in specific cases, such as pH 9 + Ca^2+^ at c_M_ = 10 wt.%. Literature on polyacrylate hydrogels indicates that Ca^2+^ binding strength depends on polymer chain length and conformation: Longer, more flexible chains can chelate Ca^2+^ bidentately, creating stronger crosslinks, whereas shorter and/or constrained chains may bind less effectively [31]. This suggests that PNAG polymer chains may also develop certain conformations that could affect the type of Ca^2+^ coordination, such as limiting the formation of strong ionic crosslinks when synthesized at certain (e.g., high) pH values. Furthermore, differences in the molecular weight between samples synthesized under different conditions could influence the effectiveness of Ca^2+^ binding. However, observations from subsequent swelling studies (Figure 6) showed that particles synthesized under acidic conditions remained more compact in acidic media than in Ca^2+^‐containing media at elevated pH. Overall, this leads to the assumption that chain conformations and flexibility might be the cause of different abilities of PNAG to form strong ionic crosslinks with Ca^2+^ (see below for multiple‐quantum nuclear magnetic resonance (MQ‐NMR) investigations).

Under acidic synthesis conditions (pH 3), Ca^2+^ had a contradictory influence on max. G′ depending on the monomer concentration: At low to medium monomer concentrations, Ca^2+^ decreased gel stiffness, whereas it increased gel stiffness at high monomer concentration, especially if synthesis is performed with low quantities of photoinitiator (Figure 3B’). One possible explanation for this observation is differences in network homogeneity. At low to medium monomer concentrations, at which the number of deprotonated carboxylate groups is limited, Ca^2+^ likely competes for these sites and forms localized clusters [32, 33]. Under these conditions, Ca^2+^ may coordinate in less stable monodentate modes rather than forming extended bidentate or bridging crosslinks [33]. These clusters, if unevenly distributed in a given volume due to the low number of carboxylates, may disturb the hydrogen bonding that is otherwise dominating, potentially disturbing the network (low G′ at low monomer concentration in the presence of Ca^2+^). At high monomer concentrations, however, the higher total number of carboxylate groups may allow Ca^2+^ to form a more homogeneous network with predominantly bidentate or bridging coordination, resulting in stiffer gels. Further studies may be needed to confirm these assumptions related to different binding modes and network heterogeneity, while here attention should be placed on microfluidic particle preparation and investigations of drug loading.

Thirdly, the evaluation of the loss factor (Figure 3C) revealed changes in the viscoelastic gel character depending on the pH of synthesis. Particularly at medium and high monomer concentrations, the contribution of the elastic component increased (tan δ decreased) with rising pH. Such enhancement of the elastic contribution can occur in materials with chain entanglements that additionally experience electrostatic repulsion of carboxylates, which is known for other anionic polyelectrolyte gels [34]. The difference in the degree of deprotonated groups between pH 3 (∼9.1%), pH 7.4 (∼99.96%), and pH 9 (∼100%), as calculated based on a p*K*_a_ of 4, suggests a dominant effect of electrostatic repulsion on tan δ. In the deprotonated state, repulsion by negative moieties forces the chains to adopt a more stretched conformation. This reduced chain flexibility, combined with tension within the entangled network, increases G′ and thereby lowers the loss factor. The difference in tan δ for materials synthesized at pH 7.4 versus pH 9 is unlikely to be driven solely by ionic effects, given the minimal difference in the degree of deprotonation between these two pH conditions. Instead, differences in polymerization/physical network formation may account for this effect. Although gelation at pH 9 was significantly delayed, likely due to reduced radical reactivity, it may result in more slowly and uniformly growing network structures, potentially resulting in a more regular gel structure with fewer internal defects.

The addition of Ca^2+^ resulted in an increased loss factor at low and moderate monomer concentrations; conversely, at high monomer concentrations, the introduction of Ca^2+^ showed a reduced loss factor, as displayed at pH 3 (Figure 3C’). This switch may, in accordance with the interpretation of Figure 3B, be explained by differences in network homogeneity, as discussed before.

In summary, the photorheology studies provided key insights for optimized microparticle synthesis. Increasing synthesis pH enhanced gel elasticity (lower tan δ, Figure 3C) due to electrostatic repulsion and chain stretching, but reduced their stiffness (lower max. G′, Figure 3B). Ca^2^^+^ partially counteracted this via ionic crosslinking, even at pH 3 (i.e., slightly below p*K*_a_), the condition with rapid polymerization. For subsequent experiments, pH 3 was chosen as the standard synthesis condition, with pH 5 as a comparison. Monomer concentrations of 10 wt.% and 25 wt.% were selected, using TPO‐NP as photoinitiator. The focus was placed on 25 wt.% because this monomer concentration provided the fastest and most robust gelation. Since the viscosity of these NAG solutions showed only minor differences compared to pure water (Figure S5), they were considered suitable for microfluidic processing.

### 2.2 Investigation of PNAG Network Structures

As illustrated in Figure 1, entanglements and reversible weak bonds (hydrogen bonds, ion complexation) are believed to stabilize PNAG chains into hydrogels. However, changes in these interactions, such as via shifts in pH, may change the architecture of the hydrogels. To gain further insight into the produced polymer network structures, including the contributions of different stabilizing mechanisms, oscillatory shear rheology and MQ‐NMR were applied, now using preformed gels. Conditions included pH 3 (below) and pH 6 (above the p*K*_a_) in the absence of Ca^2+^, as well as with Ca^2+^ (pH 6 + Ca^2+^) in the ionized state, the latter for deciphering additional stabilization by complexation.

As shown in Figure 4A, the rheological response at equilibrium swelling differed substantially between the three samples. The pH 3 samples featured the highest G′, whereas the pH 6 samples showed the lowest modulus. Although the pH 6 + Ca^2+^‐containing samples displayed the lowest equilibrium swelling ratio (Figure S3), their G′ remained below that of the pH 3 samples. To distinguish effects arising from differences in water content from those related to pH‐ and Ca^2+^‐dependent network interactions, rheological measurements were additionally performed at a fixed swelling degree of 50 (Figure 4B), corresponding to the lowest common swelling degree among the investigated conditions as defined by the pH 6 + Ca^2+^ samples. Under these conditions, G′ of the pH 3 samples remained mostly unchanged, while that of the pH 6 samples increased by approximately one order of magnitude, consistent with the reduction in swelling degree from 500 to 50. In addition, only the pH 3 samples exhibited a virtually frequency‐independent storage modulus over the investigated range, while the pH 6 and pH 6 + Ca^2+^ samples showed a more pronounced frequency dependence. This indicates ongoing relaxation processes within the observed timescale, which also overlaps with that probed by MQ‐NMR and corresponds approximately to characteristic times of 1–10 ms.

**FIGURE 4:**
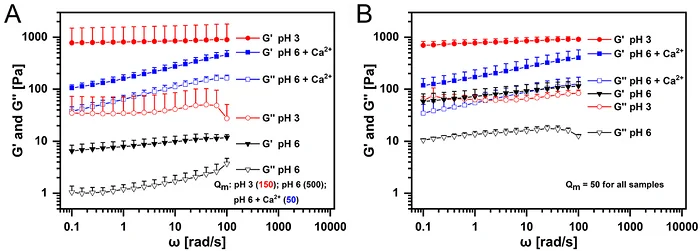
Rheology results (frequency sweeps) of the samples at (A) equilibrium swelling and (B) at the lowest common swelling degree across the investigated conditions.

The data of MQ‐NMR, which probes the interaction of nuclear spins from different nuclei and can be used to determine elastic and non‐elastic fractions within polymer networks, are shown in Figure 5. With increasing double‐quantum (DQ) evolution time, two signal intensities can be obtained: the DQ intensity (*I_DQ_*), which originates exclusively from protons in elastically active network chains, and the reference intensity (*I_ref_*), which contains the remaining signal contribution, including signals from elastically inactive components. The total signal intensity was defined as *I*_Σ*MQ*_ = *I_DQ_*  + *I_ref_*. The *I_DQ_* and *I*_Σ*MQ*_ data were fitted [35, 36]. The raw data showed that the intensity of double‐quantum coherence (two coupled nuclear spins) remained below 10% of the total signal and was rather noisy at all investigated conditions (Figure 5A), indicating that only a small fraction of protons was associated with elastically active chains. A substantial part of the signal was associated with slowly relaxing non‐elastic components. The long‐time tail in the Σ*MQ* signal can be attributed to the sol component (non‐elastic fraction 1), which includes contributions from buffer protons as well as unavoidable exchange of D_2_O with atmospheric water. This is not unexpected, as the measurements were conducted at a swelling degree of 20, at which approximately 95% of the sample consists of solvent, or 98% in the case of the Ca^2+^‐containing sample investigated at a swelling degree of 50.

**FIGURE 5:**
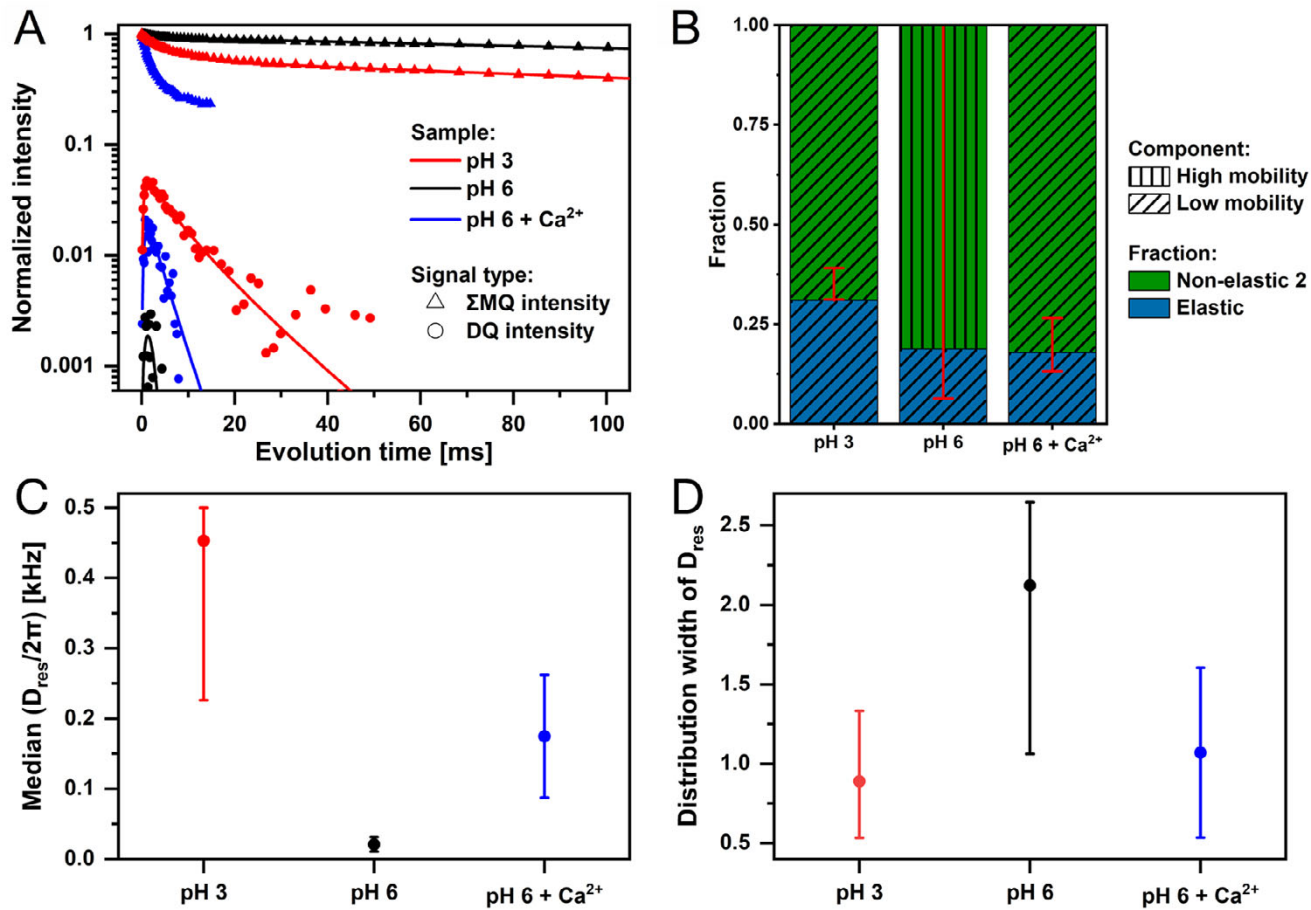
MQ‐NMR data of PNAG film samples swollen in D_2_O at defined swelling ratios: *Q_m_* = 20 for pH 3 and pH 6; *Q_m_* = 50 for pH 6 + Ca^2+^. (A) DQ build‐up and Σ*MQ* sum decay. (B) Relative polymeric component fractions obtained by simultaneous fitting after exclusion of the highly mobile sol component and normalization to the total polymeric signal. (C) Median (*D_res_*/2π) and (D) log‐normal distribution width of *D_res_* of the elastic fraction.

Fitting of the data indicated that, in addition to the isotropically mobile, non‐load‐bearing sol fraction (non‐elastic 1), the pH 3 samples contained two polymeric fractions (Figure 5B). The full component decomposition is shown in Figure S4, but the non‐elastic fraction 1 is ill‐defined, mostly because the recycle delay substantially suppressed the most mobile small‐molecule contributions with long *T_1_* relaxation time. Therefore, the component fractions shown in Figure 5B are restricted to the polymeric, non‐sol components. One polymeric component was assigned to the elastic fraction, i.e., chain segments between two different crosslinks, and accounted for about 30% of the non‐sol component. The remaining polymeric fraction, termed non‐elastic fraction 2, exhibited lower mobility than the sol fraction, which is consistent with dangling polymer chain ends or pendant loops. Upon increasing the pH to 6, the elastic fraction decreased to approximately 20%, while the non‐elastic fraction 2 showed a rather high *T_2_* value closer to that of the non‐elastic fraction 1, indicating substantially higher mobility of that fraction (see Figure S4B for data). This is in agreement with the low modulus observed in rheology for pH 6 compared to pH 3 samples (Figure 4). Upon addition of Ca^2+^, the relative elastic fraction did not clearly increase within fitting uncertainty. However, a more pronounced DQ intensity was observed, and non‐elastic fraction 2 shifted back toward lower mobility, corresponding to the less‐mobile polymeric component, similar to the pH 3 samples.

The fitted residual dipolar coupling *D_res_* parameters, which are directly related to restrictions in chain motion by crosslinks and/or entanglements, illustrated differences in chain constraints within the investigated samples. The median values (Figure 5C) indicated the weakest elastic constraints for the pH 6 samples, where electrostatic repulsion leads to increased chain mobility. In contrast, the pH 3 samples exhibited substantially stronger elastic constraints, most likely due to hydrogen‐bonding interactions resulting in a higher effective density of physical netpoints. The pH 6 + Ca^2+^ samples showed intermediate median values, although a direct quantitative comparison with the other two conditions is limited because they had to be investigated at a higher swelling degree. The distribution widths of *D_res_* (Figure 5D), which is a measure of network heterogeneity originating from different levels of local constraints that apply to different polymer segments within the network material, indicated pronounced heterogeneity. The pH 3 and pH 6 + Ca^2+^ samples exhibit similar distribution widths. In contrast, the very weak elastic contribution of the pH 6 samples resulted in a distribution width that should be interpreted with caution, because the fitting of the very small DQ signal might be prone to larger experimental errors.

Together with the swelling and rheological data, the MQ‐NMR results provide molecular‐level insight into the network constraints within the investigated samples. The finite swelling ratios, i.e., the absence of dissolution, indicate that the networks must be stabilized by constraints that persist in the long‐time limit. These constraints should mainly involve chemical crosslinks. However, contributions from stable physical interactions, including ionic interactions or extended hydrogen bonding, as observed for example in gelatin, cannot be excluded. Although the DQ signal of the pH 6 sample is very small and noisy, it should be stressed that a small permanent elastic component is reliably observed in this sample as well.

An interesting aspect is the behavior of the pH 6 + Ca^2+^ sample, which showed a lower equilibrium swelling degree (Figure S3) and higher storage modulus (Figure 4) than the pH 6 sample, indicating network stabilization by Ca^2+^. However, compared with the pH 3 sample, the pH 6 + Ca^2+^ sample still exhibited a lower storage modulus despite its lower equilibrium swelling degree. If the reduced swelling were mainly caused by additional elastically active Ca^2+^‐mediated crosslinks, a higher rather than lower storage modulus would be expected. This suggests that the reduced swelling in the presence of Ca^2+^ may not solely result from additional crosslinking, but may also involve ion‐specific swelling effects, such as reduced osmotic pressure in the presence of bivalent ions. Such an interpretation is consistent with the MQ‐NMR data, where the pH 6 + Ca^2+^ sample showed only intermediate elastic constraints compared with the pH 3 sample. Similar behavior has previously been described for polyacrylate gels, where bivalent cations reduced equilibrium swelling without an impact on crosslinking density [37]. These effects should therefore be taken into account in future studies of similar hydrogel systems.

### 2.3 Microfluidic Particle Production

The microfluidic particle preparation was performed in a coaxial glass microfluidic system, which was connected to a photoreactor (Figure 1). The photoreactor cell was composed of FEP (fluorinated ethylene propylene copolymer) tubing, a chemically inert fluoropolymer known to exhibit sufficient transparency to UV light in the relevant spectral range of TPO‐NP (385–420 nm), enabling in situ photopolymerization [24, 38]. When transferring the PNAG synthesis from model films to particle production, a stronger light scattering by dispersed droplets, as well as scattering and absorption by the wall of the microfluidic channel, may be expected. Therefore, several concentrations of the TPO‐NP photoinitiator have been evaluated in this part of the study for hydrogel particle production.

The flow rates of the individual phases are key parameters of microfluidic device operation. The combined fluid flow rate Q_total_ determined the residence time (τ) within the microfluidic photoreactor, which correlated directly with the duration of exposure of the particles to UV radiation. Here, Q_total_ was dominated by the outer phase flow rate Q_O_. Across samples, both Q_O_ and the residence time were varied (15 to 29 min) to systematically study droplet formation conditions. In all cases, the residence time was about one order of magnitude longer than required for gel formation in the rheological studies with film samples.

First, the effect of monomer phase composition and of flow rates on particle morphology was evaluated, with a focus on achieving particle homogeneity and the absence of debris, the latter being indicative of incomplete gelation. Table 1 summarizes the experimental conditions and the resulting particle diameters, while the corresponding microscopic images are provided in Figure S6.

**TABLE 1:** Conditions of microfluidic synthesis of PNAG hydrogel microparticles (MP) and resulting MP diameters d_MP_ and c (CV). Different compositions of the inner phase (concentrations c of NAG, TPO‐NP, CaCl_2_) and flow rates Q (index I: inner phase containing NAG/TPO‐NP/CaCl_2_; index O: outer phase) were investigated.

Sample code	*c*_NAG_ [wt.%]	*c*_TPO‐NP_ [wt.%]	$c_{{\mathrm{CaCl}}_{2}}$ [wt.%]	pH	*Q*_I_ [µL/h]	*Q*_O_ [µL/h]	*τ* ^a^ [min]	*d*_MP_ ^b^ [µm] ± SD	CV [%]
**MP 1.1**						2400	29	**72 ± 12**	**16.02**
**MP 1.2**	10	0.01	—	3	60	3600	20	**45 ± 9**	**19.55**
**MP 1.3**						4800	15	**27 ± 3**	**9.58**
**MP 2.1**						2400	29	**54 ± 3**	**5.10**
**MP 2.2**	25	0.001	—	3	60	3600	20	**37 ± 1**	**3.81**
**MP 2.3**						4800	15	**30 ± 4**	**15.00**
**MP 3.1**						2400	29	**15 ± 12**	**76.03**
**MP 3.2**	25	0.1	—	3	60	3600	20	**20 ± 11**	**52.15**
**MP 3.3**						4800	15	**9 ± 8**	**88.57**
**MP 4**	25	0.001	5	3	60	3600	20	**38 ± 3**	**8.84**
**MP 5*^c^***	10	0.001	5	5	60	3600	20	**43 ± 7**	**16.05**

^a^ Residence time ***τ***, calculated based on *Q*_total_ (*Q*_I_ + *Q*_o_) and volume of FEP tubing;

^b^ Diameter of synthesized particles as analyzed in the continuous oil phase by light microscopy (*n* = 100);

^c^ Monomer concentration had been reduced due to the risk of precipitation.

In the MP 1 series, which was produced with a medium monomer concentration of 10 wt.%, increasing *Q*_total_ led to particle suspensions with a visible turbidity of the suspension medium. Microscopic analysis revealed the presence of some debris around particles, indicative of incomplete gelation, particularly at shorter residence times in the UV reactor (Figure S6, MP 1.2 and 1.3). Extending UV exposure to 29 min (MP 1.1) significantly reduced debris, almost eliminating turbidity in the suspension. However, at lower flow rates (conditions for MP 1.1), an imperfect interfacial stabilization led to droplet coalescence and partial fusion of gelled particles, producing irregularly shaped samples (Figure S6, MP 1.1).

In contrast, the groups of MP 2 and MP 3 samples, prepared with higher monomer concentrations of 25 wt.%, exhibited complete gelation without turbidity or debris. However, MP 3 (high photoinitiator concentration) displayed a significantly higher coefficient of variation (CV) of particle sizes than MP 2. While TPO itself should not directly contribute to this divergence, its formulation in nanoparticles (TPO‐NP, 5% TPO) includes 95% Brij 58, a hydrophilic surfactant with a high HLB value (15.7) [39]. The presence of Brij 58 in the disperse phase apparently reduced the interfacial tension between the disperse and continuous phases, thereby increasing the Capillary and Weber numbers of the two phases, respectively. These conditions (presence of high Brij 58 content) occasionally disrupted the dripping regime, in some cases showing a transition of the microfluidic operation to jetting conditions, which compromised particle size uniformity.

Among the MP 2 samples, lower flow rates (*Q*_O_ = 2400 and 3600 µl/h) were advantageous to produce monodisperse particles at stable flow conditions. The particle size could be tuned by adjusting the flow rate, with MP 2.2 achieving high uniformity at desirably small sizes. The analysis of NAG monomer conversion by FTIR confirmed a high polymerization efficiency: MP 2.2 particles showed a conversion rate of >94%, indicating complete gelation (Figure S2).

Subsequently, in further experiments, the droplet formation in the presence of Ca^2+^ was investigated. Some of these experiments (MP 4) were conducted below the p*K*_a_ of PNAG at 25 wt.% monomer concentration and others (MP 5) above the p*K*_a_ at 10 wt.% monomer. Reducing the monomer concentration for MP 5 was necessary to prevent precipitation in the inner phase, which occurred by combination of NAG and Ca^2+^ at elevated pH. Particle generation above the p*K*_a_ (MP 5) was unstable, with particle sizes and CV comparable to MP 1.2. The MP 4 series, prepared in the presence of Ca^2+^ at pH 3, produced monodisperse particles similar to MP 2.2. However, MP 4 exhibited a higher incidence of satellite droplets, leading to an increased CV. Overall, the presence of Ca^2+^ appeared not to be advantageous for particle production.

Overall, particles from MP 2.2 conditions displayed preferable properties and low CVs (<10%), based on which they were selected for a more comprehensive characterization of stimuli‐sensitivity. Additionally, MP 4 materials were included in subsequent investigations to assess whether Ca^2+^ addition during particle synthesis influences their network structure and properties in a manner relevant to stimuli‐sensitivity.

Notably, upon isolation of the PNAG particles from the oil phase (continuous phase for particle preparation) and their transfer to an aqueous dispersion medium of the same pH value (pH 3), a substantial size increase was observed (MP 2.2: from ∼37 µm (Table 1) to ∼90 µm (Figure 6D, data at pH 3 at 0 h). This suggests that the network is not fully swollen during synthesis, which is logical since the droplets contain only a limited amount of water and are enveloped by the oil phase.

**FIGURE 6:**
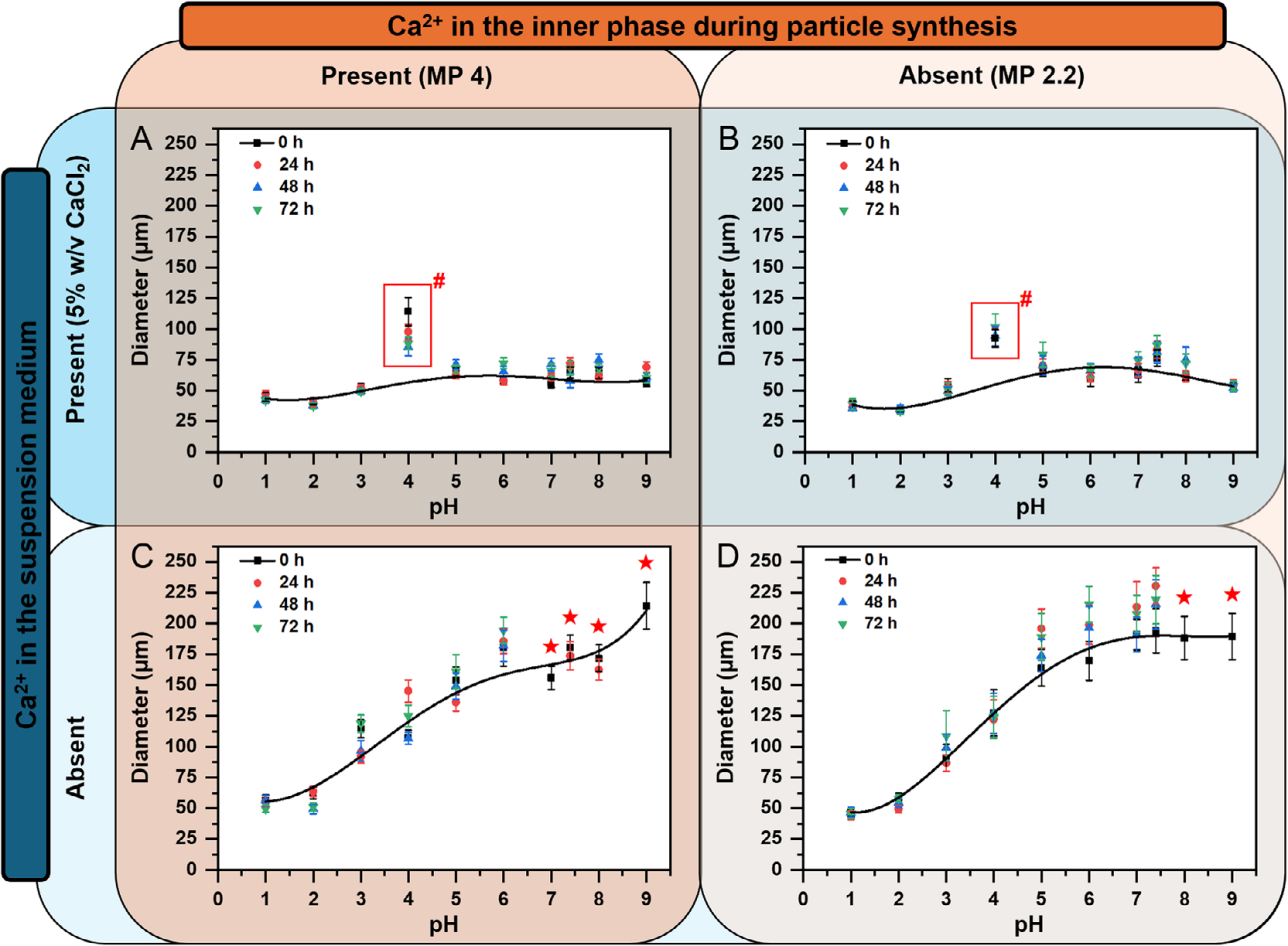
pH‐ and ion‐responsive swelling behavior of microparticles. The particles were prepared with Ca^2+^ in the inner phase (MP 4; A, C) or without Ca^2+^ (MP 2.2; B, D) and suspended in media containing 5% w/v CaCl_2_ (A,B) or lacking additional CaCl_2_ (C,D). Swelling was assessed at pH values from 1 to 9 immediately after medium exposure (0 h) as well as 24, 48, and 72 h later. Data points marked with an asterisk (^★^) indicate pH conditions where particles became invisible after the indicated time due to a lack of contrast. Red brackets with a hash symbol (^#^) highlight outliers, presumably caused by Ca^2+^ chelation by buffer ions of the specific buffer medium. Data are presented as mean ± standard deviation (*n* = 100).

### 2.4 Stimuli‐Responsive Behavior of PNAG Hydrogel Microparticles

As explained above, carboxyl groups of PNAG can, on the one hand, be deprotonated, causing ionic repulsion in bulk materials, and can, on the other hand, respond to bivalent ions by complexation and osmotic phenomena. These abilities should facilitate pH‐ and/or ion‐responsive volume switching on the level of individual particles, in principle. By using several buffers for swelling experiments, the pH value of the particles’ suspension medium was stably maintained in the range of 1–9 and monitored over the observation period, remaining within ±0.05 units of the declared pH. As shown in Figure 6, a pronounced response of particle volume to pH and the presence of Ca^2+^ could be confirmed. As expected, particle diameters increased with higher pH values, with Ca^2+^‐free media exhibiting a significantly greater swelling effect up to >5000 vol.% (Figure 6D) compared to Ca^2+^‐containing media with a swelling of approx. 200 vol.% (Figure 6B). This strong contrast highlights the role of Ca^2+^ in stabilizing the polymer network through ionic crosslinking, effectively limiting swelling. Particle sizes were repeatedly investigated at the given pH conditions over an observation period of up to 72 h, with only minimal changes in particle diameters being detected. For highly swollen particles, however, the loss of contrast to the medium presented challenges for visualization with the employed microscopic observation modes.

Interestingly, all particles exhibited similar swelling behavior in Ca^2+^‐free media, regardless of whether Ca^2+^ was present (MP 4) or absent (MP 2.2) during their microfluidic synthesis (Figure 6C,D, respectively). This observation indicates that Ca^2+^ can readily diffuse out of the polymer network during the washing steps and/or incubation. Similarly, in Ca^2+^‐containing media (Figure 6A,B), particles only swelled slightly before reaching a stable and comparable size, irrespective of whether Ca^2+^ had been supplemented during synthesis. At acidic pH, particles remained small regardless of the presence of Ca^2+^, indicating that hydrogen‐bonding interactions dominate network stabilization. At higher pH, hydrogen‐bonding interactions are increasingly replaced by Ca^2+^‐mediated ionic interactions with deprotonated groups. Although such interactions could induce further network contraction, no pronounced shrinkage was observed at higher pH. Notably, this differs from the film swelling studies, where Ca^2+^‐containing samples exhibited reduced equilibrium swelling relative to acidic samples (Figure S3), indicating that in the microparticle system Ca^2+^ mainly limits pH‐induced expansion without further network contraction. Taken together with rheological (Figure 4) and MQ‐NMR findings (Figure 5), these observations suggest that Ca^2+^‐mediated ionic interactions can modulate the network, but are not sufficiently retained to generate lasting stabilization and therefore appear to be reversible under Ca^2+^‐free conditions.

To further evaluate whether this medium‐dependent network modulation translates into reversible particle size changes, swelling and deswelling were investigated by alternating the surrounding medium between pH 3 and pH 6. The particle size changes were reversible and controllable, with particles (MP 2.2) swelling upon increasing pH and subsequently shrinking either by lowering the pH or by introducing Ca^2+^ (Figure 7). Following shrinkage, particle diameters were within the reported range of 10–100 µm for injectable depot formulations, with Ca^2+^‐induced shrinkage resulting in diameters matching the target value of approximately 50 µm. Both the pH effect and the Ca^2+^ effect were reversible, as removal of Ca^2+^ through chelation by EDTA and citrate caused particles to swell again (indicated as – Ca^2+^ in Figure 7). During swelling, the refractive‐index contrast at the particle‐medium interface decreased due to water uptake and stronger polymer‐medium interactions (Figure 7).

**FIGURE 7:**
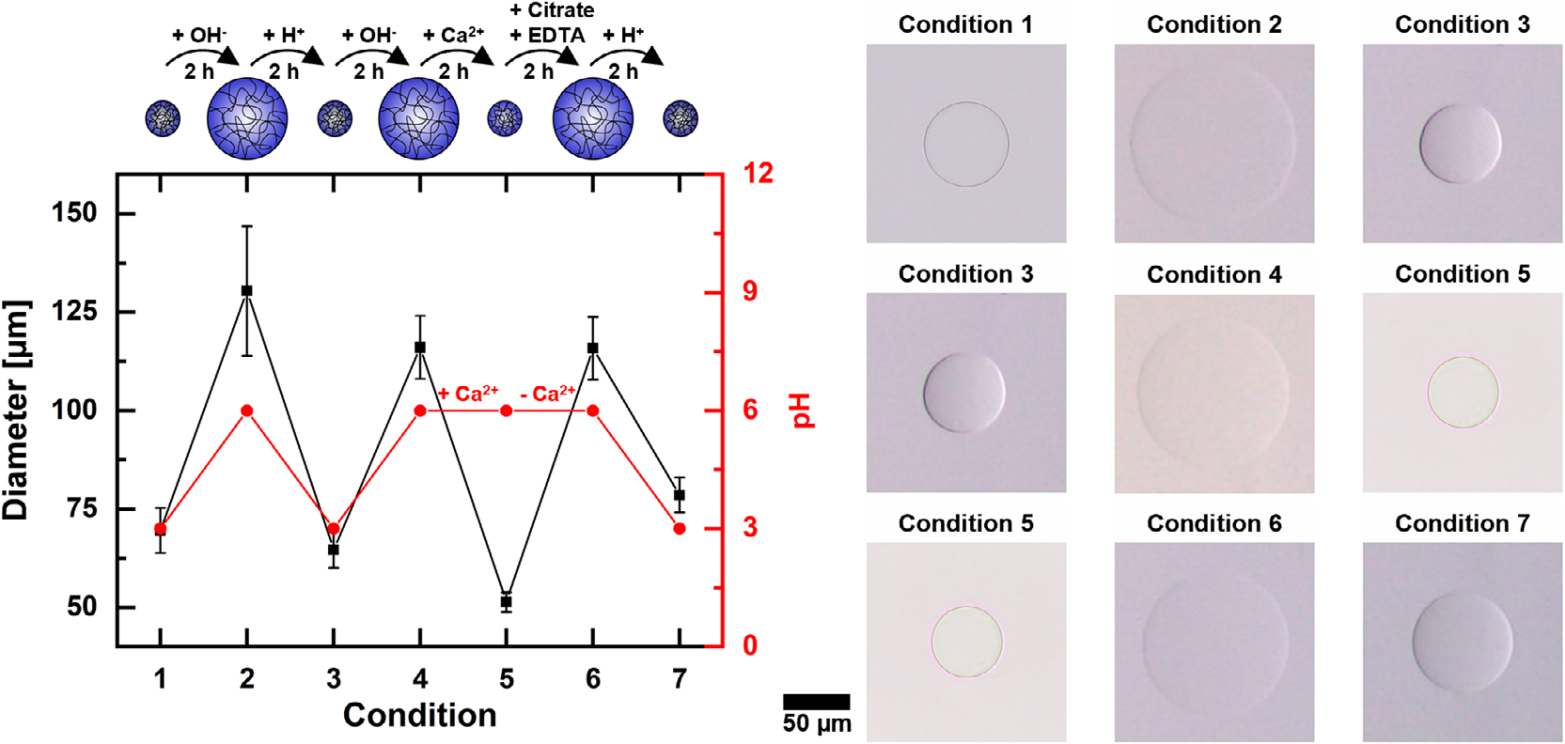
Reversible swelling and deswelling of MP 2.2 particles under varying media conditions. Left: Quantitative analysis of particle diameter and corresponding pH values under each condition. Data are presented as mean ± standard deviation (*n* = 100). Right: Microscopy images of representative particles at each condition, showcasing changes in size and contrast to the suspension medium. Scale bar: 50 µm.

The low contrast during microscopic imaging, particularly under the high‐pH conditions investigated in Figure 6 (pH > 7; pH >> p*K*_a_), raised the question of whether PNAG particles remain stable or may be subject to mass loss and dissolution. Literature suggests that the dissolution of PNAG is possible above the p*K*_a_ due to higher chain mobility in such hydrogels; for instance, the complete dissolution of larger gel structures at pH 8 within a few hours was reported [4]. In order to differentiate between particle dissolution and non‐visibility, particles suspended in Ca^2+^‐free media at pH 9 were acidified to pH 1 after 72 h. Upon acidification, particles shrank and became visible again, confirming that they had not fully dissolved despite their high surface‐to‐volume ratio compared to bulk hydrogels. The recovered particles exhibited a slightly increased diameter (54 ± 3 µm) compared to their original size (47 ± 4 µm, Figure 6D), suggesting that minor alterations in the polymer network structure may have occurred. The stability of the PNAG particles likely resulted from a polymer network containing permanent elastic constraints, which limit chain dissociation even above the p*K*_a_, as also indicated by MQ‐NMR for the pH 6 sample. The kinetics of mass loss due to particle dissolution upon prolonged exposure to elevated temperatures and higher pH may warrant future investigation.

### 2.5 Capacity for Post‐Production Loading and Release of Biopharmaceuticals

The previously observed particle swelling is associated with a loosening of the physical polymer network, which should facilitate an enhanced diffusion of drug molecules into and out of the network. Conversely, network constriction induced by the addition of Ca^2+^ can, in principle, entrap substances within the polymer network. The effectiveness of post‐production encapsulation should depend on the size of the drug molecule: it must be small enough to penetrate the polymer matrix during swelling, yet sufficiently large (or capable of interacting with polymer chains) to remain entrapped, or at least show a restricted diffusivity in the polymer mesh after particle deswelling.

In order to evaluate drug size effects on their suitability for encapsulation, different proteins/peptides were tested as model biopharmaceuticals (∼300 Da – 23 kDa). Only substances with an isoelectric point around 6 or higher were selected to avoid a net negative charge under the loading conditions. The experimental conditions and results of the post‐production loading are summarized in Table 2. To clarify the contribution of network expansion and constriction during swelling and shrinkage to loading, relative to possible net charge interactions, the swelling‐shrinkage‐based loading procedure (SML) was compared to an alternative loading procedure, direct loading (DL), in which network expansion during loading was limited by the continuous presence of Ca^2+^ as a secondary physical crosslinker.

**TABLE 2:** Loading study of PNAG microgel particles (MP 2.2).

Biopharma‐ceutical	MW [kDa]	pI	pH_Drug_	Loading Method^a^	n	Assay	Drug loading [wt.%]
**GSH**	0.307	5.93	6	SML	6	OPA	**21.34 ± 1.93**
			6	DL	4	OPA	**12.82 ± 2.06**
**Lysozyme**	14.3	11	6	SML	3	BCA	**n.d**.
**Papain**	23	8.75	6	SML	3	BCA	**n.d**.
			9	SML	6	BCA	**3.35 ± 1.26**

Data are presented as mean ± standard deviation (*n* = 3–6). Drug loading is expressed as the mass of drug per mass of dry polymer (wt.%). MW: molecular weight; pI: isoelectric point; pH_Drug_: pH of the loading solution; OPA: *o*‐phthalaldehyde assay; BCA: bicinchoninic acid assay; n.d.: no loaded model drug detectable.

^a^ Loading by SML (swelling‐mediated loading) involves swelling of freeze‐dried particles in 50 mg/ml drug solution, addition of calcium for shrinkage/drug entrapment, and washing non‐encapsulated material from the supernatant. Loading by DL (direct loading) refers to a procedure where no separate shrinkage step was applied, but Ca^2+^ was present in the medium from the beginning.

At pH 6, lysozyme and papain (pH < pI) are predominantly positively charged, which was initially expected to enhance interactions with the negatively charged polymer chains, thereby promoting accumulation and retention within the hydrogel network. However, neither lysozyme nor papain exhibited measurable loading. These results suggest that either the mesh size of the gel was too narrow compared to the hydrodynamic radii of the proteins, or the charge interactions caused a dense surface coverage of the particles by the protein, blocking further diffusion into the particle core.

To mitigate this effect, the pH can be adjusted to the pI of the proteins/peptides to achieve a state of no net charge, thereby reducing ionic interactions with the polymer and facilitating a deeper penetration into the particles. For papain, this adjustment was performed to pH 9, under which the protein and particles were incubated for ∼1 d. Afterwards, the pH was reduced to pH 6 for 1 h, followed by the addition of Ca^2+^. Under these conditions, a papain payload of 3.35 wt.% (relative to the polymer particle mass) was achieved. For lysozyme, given its high pI = 11, this experiment could not be conducted because the required pH would fall outside the applicable range and would likely compromise PNAG and lysozyme stability. Still, this evaluation shows that proteins as large as 23 kDa can penetrate into the swollen PNAG particles in principle when loaded at their pI.

Encapsulation experiments with GSH (reduced L‐glutathione), a much smaller peptide, were conducted at pH 6, which corresponds to the pI of GSH. Notably, very high loading levels of ∼21 wt.% were achieved, which is remarkable for polymer‐based drug carrier particles. Given the small size of GSH, it should be noted that a certain portion of the peptide may have been expelled along with the efflux of water during deswelling. Therefore, the GSH loading levels by the SML method were potentially even higher, and the detected quantity of 21 wt.% was already reduced compared to the original GSH uptake [40, 41]. Still, the data for GSH correspond to an almost sevenfold increase in loading levels compared to papain. This observation highlights the influence of molecule size on diffusive uptake into the hydrogel network.

In addition to the above‐discussed loading procedure with swelling and subsequent shrinkage by addition of Ca^2+^ (SML method), the loading levels of GSH were also tested when Ca^2+^ was directly added (DL method) to the loading medium, i.e., particles showed less swelling. Under these conditions, the GSH payload was strongly reduced to ∼13 wt.%. This confirms that fully opening the hydrogel network in the absence of Ca^2+^ is critical for efficient GSH diffusion into the particles. Therefore, SML was considered best suited for biopharmaceutical loading in PNAG hydrogel particles.

### 2.6 Glutathione Release Studies

At physiological pH, both GSH and the hydrogel network carry charges of the same sign (net negative), minimizing ionic interactions and making diffusion through the polymer matrix the primary mechanism of drug release. Given the dynamics of non‐covalent bonds, as seen in the complexation of Ca^2+^ with PNAG, the Ca^2+^ content in the hydrogel particles will gradually decrease over time due to exchange with the surrounding fluids. This loss of Ca^2+^ induces swelling of the polymer network, which gradually increases diffusivity. Although swelling is associated with growing diffusion path length of the payload out of the particles, the expansion of the polymer mesh during swelling apparently overcompensated for this effect. A substantial initial burst release of glutathione was observed in Ca^2+^‐free media, followed by a continuous release over several days (Figure 8). A 12‐day release profile was established for hydrogel particles loaded by the SML technique (Figure 8A). Notably, the burst release was more pronounced in DL particles (∼55 wt.%) compared to SML particles (∼40 wt.%), likely because the DL method did not provide a uniform drug distribution in the particle bulk but an accumulation in near‐surface layers due to the tighter network structure in the loading phase.

**FIGURE 8:**
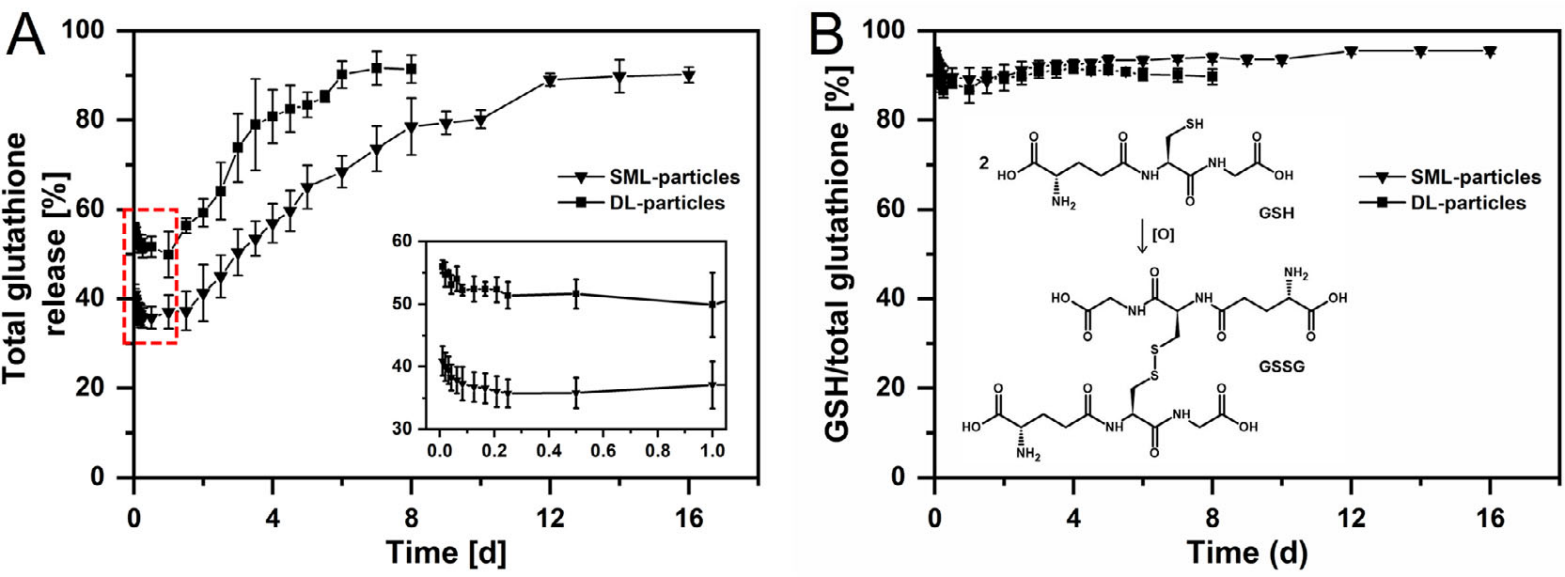
(A) Cumulative drug release behavior and (B) ratio of reduced to total glutathione in hydrogel microparticles loaded by the SML and DL methods. The structural formula in (B) illustrates the undesired oxidation reaction of reduced GSH to GSSG (instability mechanism). Data are presented as mean ± standard deviation (SML: *n* = 4; DL: *n* = 3).

To retain its therapeutic benefit as an antioxidant, glutathione must be in its reduced form (GSH) and should not be oxidized to GSSG (glutathione disulfide) during the loading and release processes. Therefore, the content of GSSG was additionally determined in the release samples. Importantly, for both loading methods (SML and DL), the ratio of GSH to total glutathione (GSH + GSSG) remained stable, with over 90% of the drug in the reduced form (Figure 8B). This emphasizes the stability of GSH in the PNAG hydrogel particles.

The investigated swelling–shrinking mechanism (SML) for drug encapsulation was confirmed to offer several advantages: Particle loading is performed post‐production, thus allowing a flexible choice of the type of payload as well as the time point of conducting the loading process according to application needs, using a stock of stable freeze‐dried particles. Compared to the DL method, SML not only enhanced the drug loading capacity but also promoted a more uniform drug distribution within the particles, eventually enabling a lower burst release and a more continuous, extended release profile.

However, some limitations of the PNAG carrier system should be mentioned, which can be a starting point for future improvements. The particles strongly tend to adhere to one another when brought into close contact, such as during centrifugation. Additionally, for a more accurate assessment of drug release that better translates to in vivo conditions, dissolution test protocols with biorelevant fluids may be added to future investigations. While Ca^2+^‐free release media were used here to challenge the carrier system's ability to retain the drug, physiological calcium levels may provide milder release conditions and further prolong the release profile.

## 3 Conclusions

This study provided insights into the effect of synthesis conditions on the properties of PNAG hydrogels. Optimal synthesis parameters were identified, allowing the successful transfer of the process to microfluidic dispersion and in‐droplet polymerization. The pH‐ and ion‐responsive behavior of the obtained PNAG particles, as well as their suitability for the encapsulation of biopharmaceuticals, was confirmed. Rheology and MQ‐NMR further linked the macroscopic pH‐ and ion‐responsive behavior to differences in elastic constraints and chain mobility within the polymer network. Furthermore, the relevance of the pI and media pH for the entry of biopharmaceuticals into the hydrogel network was illustrated and quantitatively confirmed. The swelling‐mediated loading method enabled efficient drug encapsulation under mild conditions, with remarkably high loading levels achieved for glutathione, which remained intact.

While the reported PNAG carriers show promise for drug encapsulation, more work on alternative steric stabilizers may help overcome particle aggregation, which was occasionally observed. It may also be of interest to combine NAG with other acrylate monomers, thereby tuning the overall hydrophilicity, charging, chain‐chain interactions/repulsions, and swelling of the gels. Such future work may be combined with a characterization of functional mesh sizes with probes as well as a broader investigation of loading for various therapeutically relevant biopharmaceuticals. Overall, the responsive nature of PNAG hydrogels makes them a promising candidate for further applications such as in situ tissue engineering, where controlled release of chemoattractant substances may promote tissue regeneration.

## 4 Experimental section

A detailed description of all experimental procedures and employed materials is provided in the Supporting Information.

Model studies for polymer network characterization: NAG was synthesized based on previously reported methods [4, 6] and characterized by FTIR and ^1^H‐NMR spectroscopy. The gelation behavior and product properties were determined during polymerization using oscillatory rheological measurements with a MCR 302e rheometer (Anton Paar, Graz, Austria) equipped with a cone‐plate geometry and UV light curing module (Omnicure S2000; irradiation: 250–400 nm; 10 W/cm^2^) at different pH, monomer concentration, and photoinitiator type and concentration. Additionally, preformed films were studied by oscillatory rheology with plate‐plate geometry and by swelling studies in aqueous buffer for determining the swelling ratio at different pH/ion concentration. MQ‐NMR (20 MHz Bruker MiniSpec mq20) was performed after sample drying and rehydration in D_2_O, applying an artefact‐compensated variant of the Baum‐Pines pulse sequence, as described previously [42], followed by data fitting.

Microgel preparation, characterization, and loading: Microfluidic devices were constructed from glass capillaries, operated with pulse‐free pressure‐driven pumps (OB1, ElveSys), monitored with a high‐speed camera system (PreciGenome), and used for NAG droplet generation with online photopolymerization (Omnicure S2000) to PNAG. Swelling studies in different aqueous media with intermediate media exchange and light microscopic monitoring (DSX1000, Evident Corporation) were applied to explore the dual stimuli‐sensitivity of swelling responses. For drug loading, lyophilized particles were incubated in model drug solutions above the p*K*_a_ either in the presence of 5% Ca^2+^ (direct loading) or in its absence, the latter followed by Ca^2+^ addition to induce drug capture by shrinking (swelling‐mediated loading). The non‐encapsulated drug was removed by washing steps, and loading levels were determined by a series of Ca^2+^ extraction and dialysis steps. The BCA assay (protein) and the OPA assay (GSH) were used to determine drug concentrations. Release studies for GSH were performed at 37°C after loading GSH into lyophilized particles by swelling, separation of non‐encapsulated drug by centrifugation and washing, and initiating drug release by media change to Ca^2+^‐free conditions, with analysis of drug in the supernatant.

## Author Contributions

**Yusuf Dursun**: conceptualization, methodology, formal analysis, investigation, writing – original draft, writing – review & editing, visualization. **Burak Aydin**: formal analysis, investigation, writing – review & editing, funding acquisition. **Bidit Lamsal**: methodology, formal analysis, investigation, writing – original draft. **Deniz Ceylan**: conceptualization, writing – review & editing, project administration. **Kay Saalwächter**: conceptualization, formal analysis, writing – review & editing, project administration. **Christian Wischke**: conceptualization, methodology, resources, writing – review & editing, visualization, supervision, project administration, funding acquisition.

## Conflicts of Interest

The authors declare no conflicts of interest.

## Supporting information




**Supporting File**: smll75268‐sup‐0001‐SuppMat.pdf.

## Data Availability

The data that support the findings of this study are available from the corresponding author upon reasonable request.
