# Supplementary material for: Dual‐Stimuli Responsive Poly(N‐acryloyl glycine) Microgels: Investigation of Network Structures and Application for Loading and Release of Biologics
Source: Small. 2026 Aug 20;22(55):e75268. doi: 10.1002/smll.75268 (PMC13630401; doi:10.1002/smll.75268)
Supplement: Supplementary file 1 [file SMLL-22-e75268-s001.pdf]

Supporting Information

Dual-stimuli responsive poly(*N*-acryloyl glycine) microgels: Investigation of network structures & application for loading and release of biologics

Yusuf Dursun[1], Burak Aydin[2], Bidit Lamsal[3], Deniz Ceylan[2], Kay Saalwächter [3], Christian Wischke[1,*]

[1] Institute of Pharmacy, Martin-Luther-University Halle-Wittenberg, Wolfgang-Langenbeck-Str. 4, 06120 Halle, Germany

[2] Faculty of Pharmacy, Bezmialem Vakıf University, 34093 Fatih, Istanbul, Turkey

[3] Institute of Physics, Martin-Luther-University Halle-Wittenberg, Betty-Heimann-Str. 7, 06120 Halle, Germany

*Correspondence: christian.wischke@pharmazie.uni-halle.de

1 Materials and Methods

1.1 Materials

Glycine (≥99%), acryloyl chloride (≥97%), tetrahydrofuran (≥99%, containing 250 ppm butylated hydroxytoluene), anhydrous sodium sulfate (99.0–100.5%), diphenyl(2,4,6-trimethylbenzoyl)phosphine oxide nanoparticles (TPO-NP, containing 5% TPO), anhydrous sodium hydroxide (≥98%), hydrochloric acid (37%), 2-(4-morpholino)ethanesulfonic acid (MES) monohydrate (≥99%), mineral oil, reduced L-glutathione (GSH, ≥98%), lysozyme (activity 70,000–140,000 U/mg), and dimethyl sulfoxide-d6 (DMSO-d6, containing 0.03% tetramethylsilane) were purchased from Sigma-Aldrich/Merck KGaA (Darmstadt, Germany).

Lithium phenyl-2,4,6-trimethylbenzoylphosphinate (LAP, ≥97%) and 2-hydroxy-2-methylpropiophenone (Darocur® 1173 DC1173, 98%) were purchased from VWR International (Pennsylvania, USA). Papain (> 95%, activity ≥ 1,000 TU/mg), N,N-bis(2-hydroxyethyl)-2-aminoethanesulfonic acid (BES, ≥99%), 2-(cyclohexylamino)ethanesulfonic acid (CHES, ≥98%), formic acid (≥98%), ethyl acetate (≥99.8%), ethylenediaminetetraacetic acid (EDTA) disodium salt dihydrate (≥99%), trisodium citrate dihydrate (99%), 1,2-phthaldialdehyde (OPA, 98%), Tris(2-carboxyethyl)phosphine hydrochloride (TCEP, 98%), N-Ethylmaleinimid (NEM, ≥99%), and Pierce™ BCA Protein Assay Kit were obtained from Thermo Fisher Scientific (Massachusetts, USA).

Span™ 80, calcium chloride dihydrate ($CaCl_2 \cdot 2H_2O$, ≥99%), Methanol (99.5%), and acetic acid (100%) were purchased from Carl Roth (Karlsruhe, Germany). Petroleum ether (boiling point 40-60 °C) was obtained from ORG Laborchemie (Bunde, Germany). Sodium chloride (99.0 – 100.5%) was purchased from Th. Geyer (Renningen, Germany). Boric acid (99.0 – 100.5%) was obtained from Riedel-de

Haën, Honeywell (North Carolina, USA). Ultrapure water was obtained from a TKA MicroPure filtration system (Niederelbert, Germany). All other chemicals and reagents used in this study were of analytical grade.

1.2 Methods

1.2.1 Synthesis of *N*-acryloyl glycine (NAG)

NAG was synthesized based on previously reported methods [1, 2] with some modifications. Briefly, glycine (20.04 g) was dissolved in 214 mL ultrapure water in a three-neck flask and cooled in an ice bath. Sodium hydroxide (11.86 g) was added, and the mixture was stirred until fully dissolved and cooled. A solution of acryloyl chloride (23.8 mL) in tetrahydrofuran (40 mL) was added dropwise while maintaining the pH between 7 and 8 by the addition of NaOH pellets. After completion of the reaction, the pH of the mixture was raised to pH 10 with NaOH, and the mixture was extracted twice with 60 mL ethyl acetate to remove impurities. The aqueous solution was then acidified to pH 2 with 6 M HCl and extracted five times with 400 mL ethyl acetate. The combined organic layers were dried over sodium sulfate overnight, filtered, and concentrated using a rotary evaporator. The concentrate was precipitated in cooled petroleum ether, filtered, and vacuum-dried to constant mass.

1.2.2 Kinetic analysis of photoinduced gelation

The gelation behavior was determined using oscillatory rheological measurements with an MCR 302e rheometer (Anton Paar, Graz, Austria) equipped with a UV light curing module. A 25 mm stainless steel cone-plate geometry (1° cone angle, 52 µm truncation gap) was used. Three different photoinitiators (LAP, DC1173, and TPO-NP) were investigated at varying concentrations, along with different monomer concentrations at acidic conditions (pH 3). After identifying the optimal photoinitiator, further investigations were conducted near neutral (pH 7.4) and basic conditions (pH

9) adjusted with 6 M HCl or 6 M NaOH solution, with or without the presence of Ca^{2+} (5 wt.% $CaCl_2$).

The storage modulus (G') and loss modulus (G'') were measured in strain-controlled mode at 10 Hz frequency and 10% strain at 25 °C. 60 s after measurement initiation, UV light (10 W/cm^2, 250-450 nm) was applied in situ using the OmniCure® S2000 Elite (Excelitas Technologies Corp., Pennsylvania, USA) with a High-Power Fiber Light Guide, positioned 50 mm below the sample. All measurements were performed in triplicate. The gelation behavior was assessed through three rheological parameters: (i) gelation kinetics, represented by the gel point (crossover where G' surpasses G''); (ii) gel stiffness, indicated by maximum G'; and (iii) gel quality, reflected by the loss factor (tan δ).

1.2.3 Design and operation of microfluidic droplet devices

Glass microfluidic devices were fabricated with some refinements to standard protocols [3], using two cylindrical micropipettes coaxially aligned within a square capillary (inner dimensions: 1.1 x 1.1 mm, Hilgenberg GmbH, Germany). The devices were assembled on microscope slides to provide stability. The micropipettes were prepared from glass capillaries (inner diameter: 0.58 mm; outer diameter: 1.0 mm; Science Products GmbH, Germany) using a micropipette puller (PUL-1000, World Precision Instruments, Florida, USA). The tip orifices were manually ground to outer diameters of 50 µm for the feeding pipette and 300 µm for the collecting pipette. Capillaries were plasma-treated using a plasma cleaner (PDC-002, Harrick Plasma, New York, USA) and subsequently silanized using either octadecyltrimethoxysilane for hydrophobic treatment or 2-[methoxy(poly-ethyleneoxy)propyl]trimethoxysilane (6-9 PEG units) for hydrophilic treatment. Pipettes were aligned with a 25 µm gap using

a micromanipulator and stabilized with a UV-curable glue (Ber-Fix, Germany). Blunt cannulas were added to the ends of the glass capillaries to provide three separate inlets. Epoxy glue was used to seal the system, ensuring stability and preventing leaks under elevated pressure.

The dispersed phase, consisting of an aqueous monomer solution (NAG; 10 or 25 wt.%) containing a photoinitiator (TPO-NP; 0.001, 0.01, or 0.1 wt.%) at pH 3 or 5 with or without the presence of Ca^{2+} (5 wt.% $CaCl_2$), was fed through the feeding capillary. The continuous phase, composed of mineral oil containing 3 wt.% Span™ 80, was supplied through both sides of the square capillary. Flow rates were precisely controlled using an OB1 MK3+ pressure-based flow control system with thermal flow sensors (ElveSys, Paris, France). Droplet formation at the junction was monitored in real-time using a high-speed imaging system (PreciGenome, California, USA).

Subsequent polymerization of NAG within the individual droplets was carried out in an attached reaction unit consisting of 2.5 m of spiral-wound fluorinated ethylene propylene copolymer (FEP) tubing (inner diameter: 0.8 mm; outer diameter 1.6 mm; Carl Roth, Germany). Droplets were exposed to UV light at a power density of 10 W/cm^2 using a High-Power Fiber Light Guide positioned 10 cm above the tubing. The light guide was connected to the OmniCure® S2000 Elite UV curing system, and the entire reaction unit was enclosed in aluminium foil. After polymerization, the resulting particles were continuously collected at the outlet of the FEP tubing in a beaker and stored until further processing.

1.2.4 Particle characterization

Optical characterization of microparticles was performed using light microscopy (DSX1000, Evident Corporation, Tokyo, Japan). Image analysis was conducted with

the manufacturer’s software, PRECIV Desktop 1.2 (Evident Corporation, Tokyo, Japan), using Feret’s diameter as the descriptive parameter. For each measurement, the diameters of 100 particles were recorded.

To assess the swelling behavior of microparticles, their diameters were monitored microscopically over three days in separate experiments with different buffer media spanning pH 1-9, with or without Ca^{2+} (5% w/v $CaCl_2$). The media compositions are detailed in Table S1 (Supporting Information, SI). Microparticles were initially washed by adding medium, centrifuging at 3000 g for 3 minutes, and repeating the process twice. After washing, particles were imaged immediately and at 24-hour intervals for a total of three days at room temperature. Between imaging, microparticles were stored at 4 °C and equilibrated to room temperature for 30 minutes before measurement.

To characterize the reversibility of stimuli-induced swelling-deswelling, microparticles that had been freeze-dried from a pH 1 solution were rehydrated in buffer (0.1 M formic acid and 0.1 M MES) and adjusted to pH 3. The pH was then adjusted between 3 and 6 by stepwise addition of 6 M NaOH or 6 M HCl solution. The effects of Ca^{2+} ions on swelling-deswelling were investigated by adding $CaCl_2$ to a final concentration of 5% w/v. The effect of Ca^{2+} was subsequently reversed by chelation using a pH 6 buffer containing 0.1 M EDTA and 0.2 M citrate. After each adjustment, particles were allowed to equilibrate for at least 2 hours.

1.2.5 Conversion of NAG to PNAG during particle synthesis

Attenuated total reflection-Fourier transform infrared spectroscopy (ATR-FTIR) was used to confirm the successful synthesis of NAG and PNAG. Freeze-dried PNAG microparticles obtained from a pH 1 solution were used as the PNAG sample. ATR-FTIR spectra were recorded using a VERTEX 70 spectrophotometer (Bruker Optics, Massachusetts, USA) equipped with a horizontal attenuated total reflectance device

featuring a diamond crystal. Spectra were collected over a range of 4000 to 400 cm^{-1}. The degree of conversion of NAG to PNAG was evaluated semi-quantitatively. Two signals were of interest: the peak at 804 cm^{-1}, corresponding to C=C bending, was used for evaluating the monomer conversion, and the peak at 1548 cm^{-1}, corresponding to N-H bending, was used as an internal standard for normalization of the spectra.

Proton nuclear magnetic resonance (^1H NMR) spectroscopy was used to confirm the successful synthesis of NAG. ^1H NMR spectra were recorded at 400 MHz using a Varian VNMRS spectrometer (Agilent Technologies, California, USA). DMSO-d6 containing 0.03% tetramethylsilane was used as the solvent for NAG. The resulting spectra are depicted in Figures S1 and S2.

1.2.6 Characterization of film samples by equilibrium swelling, rheology and solid-state NMR

Samples used for these investigations were presynthesized pristine PNAG hydrogel films. The films were prepared in Petri dishes from a 25 wt.% NAG solution at pH 3 containing 0.001 wt.% TPO-NP photoinitiator, using a solution height of 2 mm. Polymerization was performed by UV irradiation at 10 W/cm^2 for 30 min. The resulting hydrogel films were subsequently soaked in ultrapure water and then in 0.1 M HCl solution at pH 1. After removal of excess surface liquid, the films were lyophilized from the pH 1 state.

Swelling experiments were first performed to determine the equilibrium swelling degree of the hydrogel films. For this purpose, dried hydrogel films were cut and placed into polyester nets to facilitate handling of the soft hydrogel samples. The dry masses

of the film (m_d) and the polyester net (m_N) were determined prior to swelling. The hydrogel films were then placed in media of pH 3, pH 6, and pH 6 + Ca^{2+} (for details, see Table S1), representing conditions below the pK_a of PNAG, above the pK_a of PNAG, and above the pK_a in the presence of Ca^{2+} ions, respectively. At predefined time points, the nets were removed from the medium, allowed to drain for 30 s, and gently blotted with lint-free wipes to remove excess surface liquid before weighing.

The swelling degree was defined as the mass-based swelling ratio Q_m:

$$Q_m = \frac{m_t - m_N}{m_d}$$

where m_t is the mass of the swollen sample together with the polyester net at time t, m_N is the mass of the dry polyester net, and m_d is the dry mass of the hydrogel film.

Oscillatory shear rheology measurements on the hydrogel films were performed with the MCR 302e rheometer (Anton Paar, Graz, Austria) equipped with a standard plate module. A 25 mm (for pH 3 and pH 6 + Ca^{2+}) or 50 mm (for pH 6) stainless steel plate-plate geometry setup was used. Samples taken from the swelling study were characterized both at their respective equilibrium swelling degree and, after lyophilization and rehydration, at a fixed swelling degree of Q_m = 50, corresponding to the lowest common swelling degree, to remove the effect of swelling-related chain stretching on the modulus for comparison.

The storage modulus (G') and loss modulus (G'') were first measured in an amplitude sweep at an angular frequency of 10 rad/s to determine the linear viscoelastic region. Subsequently, frequency sweeps were performed using strain amplitudes within the linear viscoelastic region, ranging from 1 to 5% depending on the sample.

For MQ-NMR, the same samples as for rheology were used, which involved lyophilization and reswelling in D_2O to a swelling degree of Q_m = 20. For the calcium-containing sample, salt precipitation was observed; hence, this sample was investigated at a swelling degree of Q_m = 50. MQ-NMR was used to probe the residual dipolar coupling constant (D_{res}) of protons in the polymer network chains. D_{res} reflects the degree of anisotropy in the orientation fluctuations of repeating units within the polymer chains and thereby provides information on the density of crosslinks and topological constraints within the network, i.e., the single-chain elasticity. In contrast, elastically inactive defects, such as dangling chains, as well as sol fractions, exhibit $D_{res} \approx 0$ due to isotropic tumbling. The samples were measured using a 20 MHz Bruker MiniSpec mq20, applying an artefact-compensated variant of the Baum-Pines pulse sequence, as described previously [4]. The 90° pulse length was 2.1 μs, and the recycle delay was adjusted between 1 and 3 s depending on the longitudinal relaxation time of the network fraction.

With increasing double-quantum (DQ) evolution time, two signal intensities can be obtained: the DQ intensity (I_{DQ}), which originates exclusively from protons in elastically active network chains, and the reference intensity (I_{ref}), which contains the remaining signal contribution, including signals from elastically inactive components. The total signal intensity was defined as $I_{\Sigma MQ} = I_{DQ} + I_{ref}$.

The I_{DQ} and $I_{\Sigma MQ}$ data were fitted according to a previously described procedure [5], with modifications. The present data were best described by a model consisting of two defect fractions and one network fraction with a log-normal distribution of D_{res}, while minimizing the number of fitting parameters. These fractions were denoted as non-elastic 1 (longest T_2), non-elastic 2 (shorter T_2), and network, respectively. The error

bars, reflecting the fitting uncertainty, were determined from χ^2 maps of the relevant fitted parameters, namely the network fraction, the residual coupling D_{res}, and the width parameter σ of the log-normal D_{res} distribution, following the procedure described in [6]. Here, χ^2 was used as a goodness-of-fit measure for the agreement between the experimental data and the fitted model; its calculation was performed using the standard implementation of the Python package lmfit [7].

1.2.7 Drug loading

Drug loading was assessed using MP 2.2 microparticles (Table 1), which were freeze-dried from a pH 1 solution. Two encapsulation approaches were investigated:

(i) In the first approach, termed "swelling-mediated loading" (SML), 30 mg of lyophilized particles were suspended in a 50 mg/ml drug solution buffered above the pK_a of PNAG, to induce swelling. The suspension was agitated for 24 hours on a GFL 3006 horizontal shaker (GFL, Burgwedel, Germany) at 50 rpm. Subsequently, 20% w/v $CaCl_2$ was added to a final concentration of 5% w/v, inducing deswelling (shrinkage) of particles. To remove non-encapsulated drug, particles were thoroughly washed with a 5% w/v $CaCl_2$ solution. Drug extraction (determination of loading levels) was performed by adding a buffer solution capable of binding Ca^{2+} through precipitation and/or chelation. For dialyzable payloads (papain and lysozyme), 0.1 M CHES buffer containing 0.1 M EDTA, 0.2 M citrate, and 0.5 M NaCl was used. For GSH, which is non-dialyzable due to its low molar mass, a 0.1 M phosphate buffer with 50 mM citrate was directly added to the sample. The suspension was shaken for 3 hours. After centrifugation, the supernatant was collected, and this extraction process was repeated twice to ensure complete recovery of the drug.

(ii) For samples that achieved a noteworthy loading level with the SML method, a second approach, termed "direct loading" (DL), was examined for comparison. In the

DL method, the swelling step in the absence of Ca^{2+} was omitted. The lyophilized particles were directly immersed in a drug solution at elevated pH, in which $CaCl_2$ was added (5% w/v), keeping the polymer network in a constricted state. All other handling and experimental procedures were identical to those of the SML protocol.

The quantification of the payload was carried out using either an *o*-phthalaldehyde (OPA) assay (for GSH) or a bicinchoninic acid (BCA) assay (for papain and lysozyme). Liquid samples, including the supernatant and washing solution from drug loading experiments as well as the medium from release studies, were investigated.

For the OPA assay, 100 µL of the samples were mixed with 25 µL 10 mM TCEP and incubated for 15 minutes. Next, 125 µL of a 1 mg/ml OPA solution in borate buffer (containing 10 vol.% methanol) was added, and the mixture was incubated at 37 °C for an additional 15 minutes. Fluorescence was measured with an excitation wavelength of 340±10 nm and an emission wavelength of 450±10 nm.

For the BCA assay, samples were first dialyzed to remove interfering components such as EDTA, citrate, and NaCl, while retaining the proteins. Subsequently, 25 µL of the dialyzed samples were mixed with 200 µL of the BCA reagent and incubated at 37 °C for 30 minutes. The absorbance was measured at 562 nm. All measurements were performed using a BioTek Cytation 5 (Agilent, California, USA).

1.2.8 In vitro drug release

Release studies were performed in 15 mL centrifuge tubes. First, GSH was loaded into 30 mg lyophilized particles using both SML and DL methods as described previously. The medium was supplemented with 0.01% w/v Tween® 80 to minimize particle aggregation during centrifugation, which was necessary to separate loaded particles from the supernatant. The pH of the washing solution during this work-up was

7.4 using 0.05 M BES buffer. The total glutathione payload of the particles was determined by measuring the remaining GSH concentration in the soaking and washing solutions and subtracting this value from the initially added amount. The quantification of GSH was conducted using the OPA assay, following the protocol described above.

The drug release was initiated by adding 8 mL of Ca^{2+}-free 0.05 M BES buffer (pH 7.4) to the freshly loaded particles, bringing the total aqueous volume to 10 mL. Samples were incubated at 37 °C in a shaking water bath (GFL 1083, GFL, Burgwedel, Germany). At predetermined time points, duplicate aliquots (500 µL each) for independent analyses were withdrawn from the supernatant of each sample and replaced with an equal volume of pre-warmed fresh buffer.

To distinguish between reduced glutathione (GSH) and oxidized glutathione, i.e., glutathione disulfide (GSSG), duplicate aliquots were analyzed separately. One aliquot was directly reduced with TCEP and used to determine the total glutathione content (GSH + GSSG). The second aliquot was first treated with 10 mM N-ethylmaleimide (NEM; volume adjusted according to the sampling interval to maintain a stoichiometric excess), thereby alkylating GSH and preventing its further oxidation. In this way, only the fraction originally present as GSSG in the second aliquot became accessible for quantification after subsequent reduction with TCEP. The GSH fraction was then calculated from the difference between the total glutathione content and the GSSG fraction, allowing determination of the ratio of GSH to total glutathione. All sample handling was performed under inert conditions.

2 Supporting results

Table S1: Compositions of media used to evaluate the pH- and Ca^{2+}-dependent swelling behavior of poly(N-acryloyl glycine) (PNAG) hydrogel microparticles. Media spanning pH 1–9, with and without the presence of Ca^{2+} (5% w/v $CaCl_2$).

pH value	Media
**1**	0.1 M hydrochloric acid
**2**	Modified buffer solution pH 2.0 (based on Ph. Eur.), containing 88.13 mmol/L NaCl and 11.9 mmol/L HCl
**3**	0.1 M formate buffer
**4**	
**5**	0.1 M acetate buffer
**6**	0.1 M MES buffer
**7**	0.1 M BES buffer
**7.4**	
**8**	
**9**	0.1 M CHES buffer

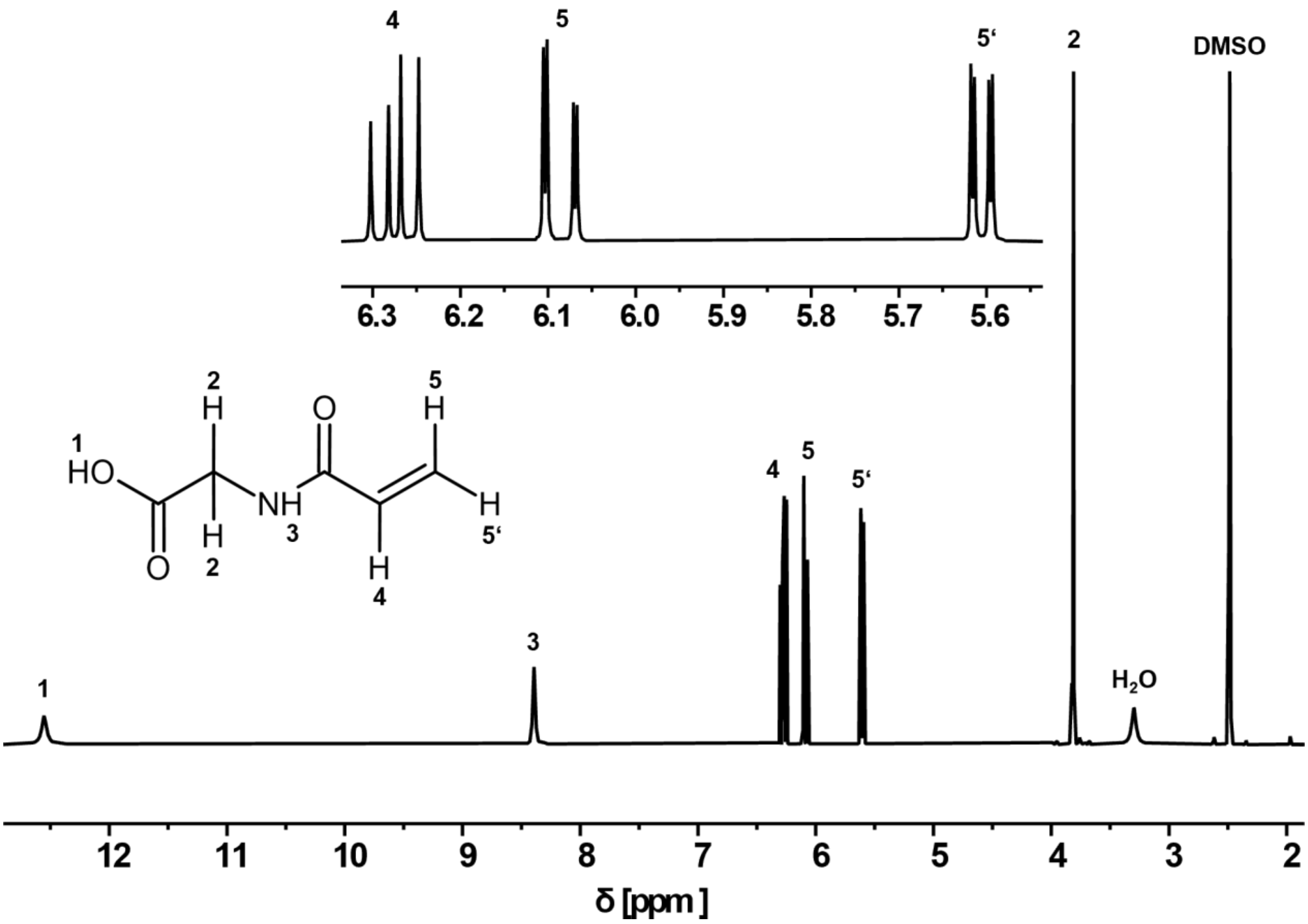

Figure S1: Proton nuclear magnetic resonance (^{1}H-NMR) spectrum of synthesized N-acryloyl glycine (NAG) monomer recorded in DMSO-d_6. Chemical shifts (δ) are labelled as follows: H1 (12.6 ppm, hydroxyl proton), H2 (3.8 ppm, glycine methylene protons), H3 (8.4 ppm, amide proton), H4 (6.3 ppm, secondary vinyl proton), H5 (6.1 ppm, terminal vinyl proton), and H5′ (5.6 ppm, terminal vinyl proton). The inset highlights the resonances of the secondary and terminal vinyl protons (H4, H5, and H5′). The molecular structure of NAG with assignments is depicted for reference.

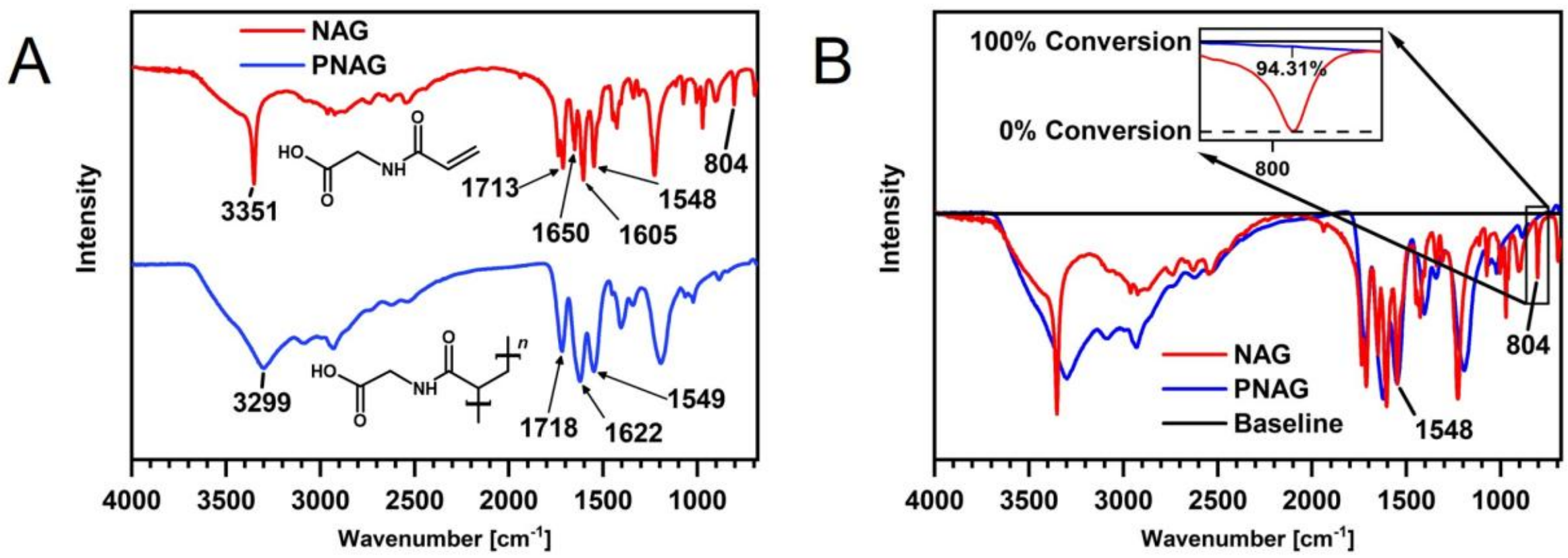

Figure S2: ATR-FTIR spectra of synthesized NAG monomer powder (red) and lyophilized PNAG microparticles (blue), recorded in the range of 4000–400 cm^{-1}. (A) Characteristic bands for NAG include N–H bending at 1548 cm^{-1}, N–H stretching at 3351 cm^{-1}, C=C bending at 804 cm^{-1}, C=C stretching at 1605 cm^{-1}, and C=O stretching at 1650 cm^{-1} and 1713 cm^{-1}. In the PNAG spectrum, similar peaks were observed, with shifts due to polymerization: N–H bending at 1549 cm^{-1}, N–H stretching at 3299 cm^{-1}, and C=O stretching at 1622 cm^{-1} and 1718 cm^{-1}. The molecular structures of NAG and PNAG are depicted for reference. (B) Overlay of spectra to determine the degree of conversion. Spectra were matched by aligning their baselines and normalized using the constant peak at 1548 cm^{-1} (N-H bending). The intensity at 804 cm^{-1} (C=C bending) was used to evaluate the monomer conversion. The inset provides a magnified view of the change in intensity of the analyzed signal, based on which the degree of conversion was calculated according to:

$$Conversion\ [\%] = \left(\frac{I_{PNAG,804\ cm^{-1}} - I_{NAG,804\ cm^{-1}}}{I_{baseline} - I_{NAG,804\ cm^{-1}}}\right) \times 100\%\ ;\ I = Intensity.$$

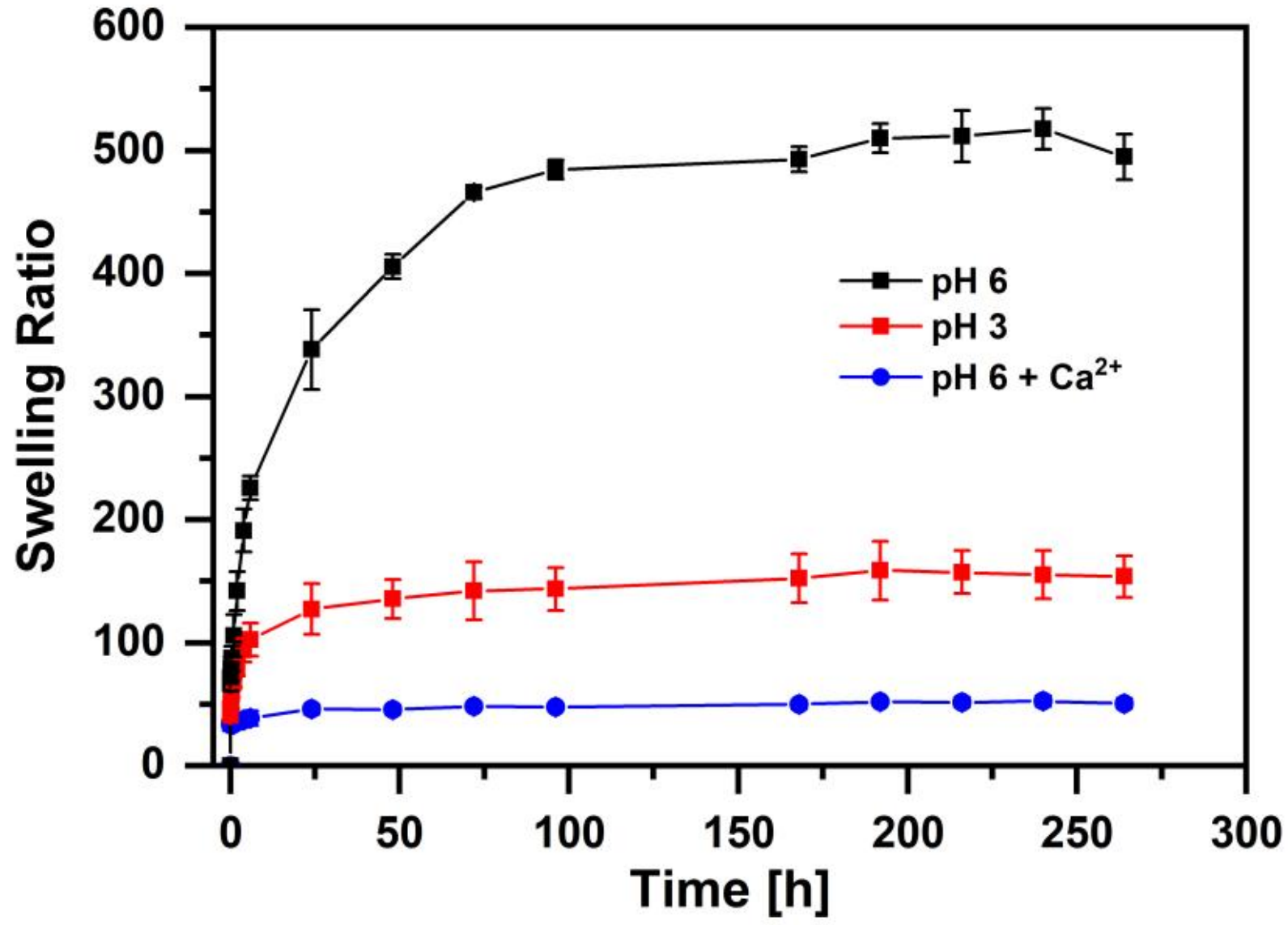

Figure S3: Swelling kinetics of pristine PNAG hydrogel films in different media: formate buffer (pH 3), MES buffer (pH 6), and MES buffer containing 5% w/v $CaCl_2$ (pH 6 + Ca^{2+}). Equilibrium swelling ratios of approximately 150, 500, and 50 were obtained for the pH 3, pH 6, and pH 6 + Ca^{2+} samples, respectively.

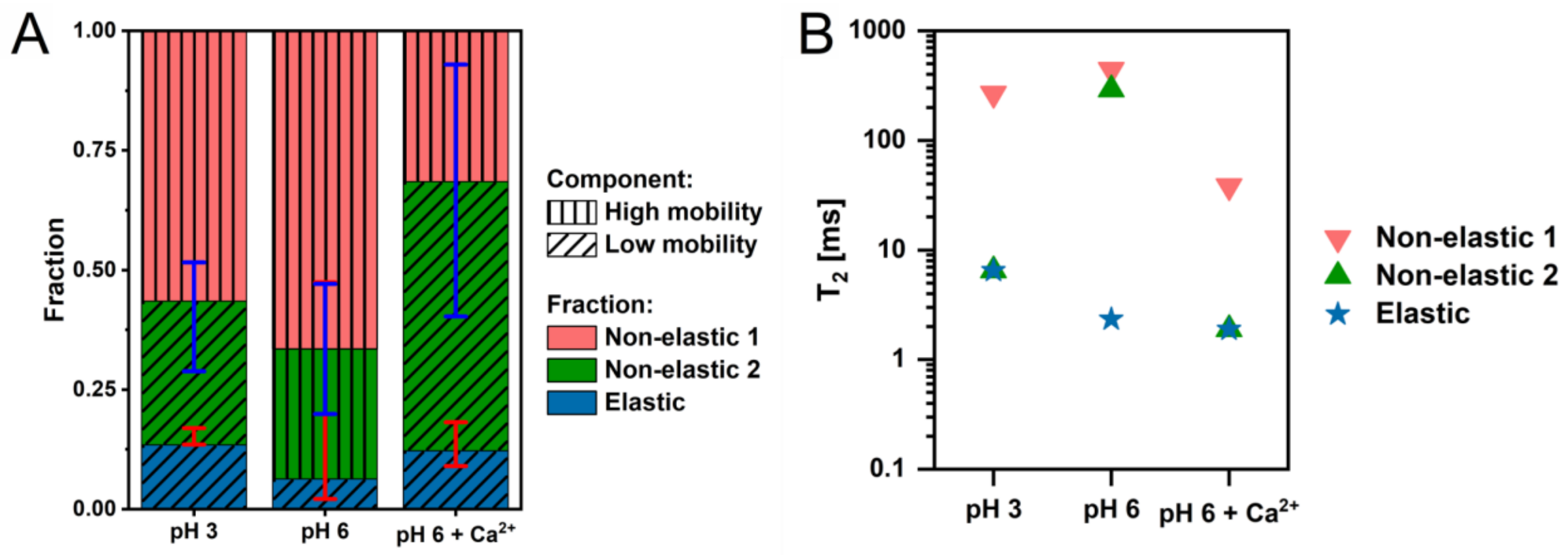

Figure S4: MQ-NMR data of PNAG film samples swollen in D_2O at defined swelling ratios: Q_m = 20 for pH 3 and pH 6; Q_m = 50 for pH 6 + Ca^{2+}. (A) Component fractions obtained by simultaneous fitting of the intensity curves, including the highly mobile non-elastic fraction 1 shown for completeness only. (B) T_2 relaxation times of the fitted components.

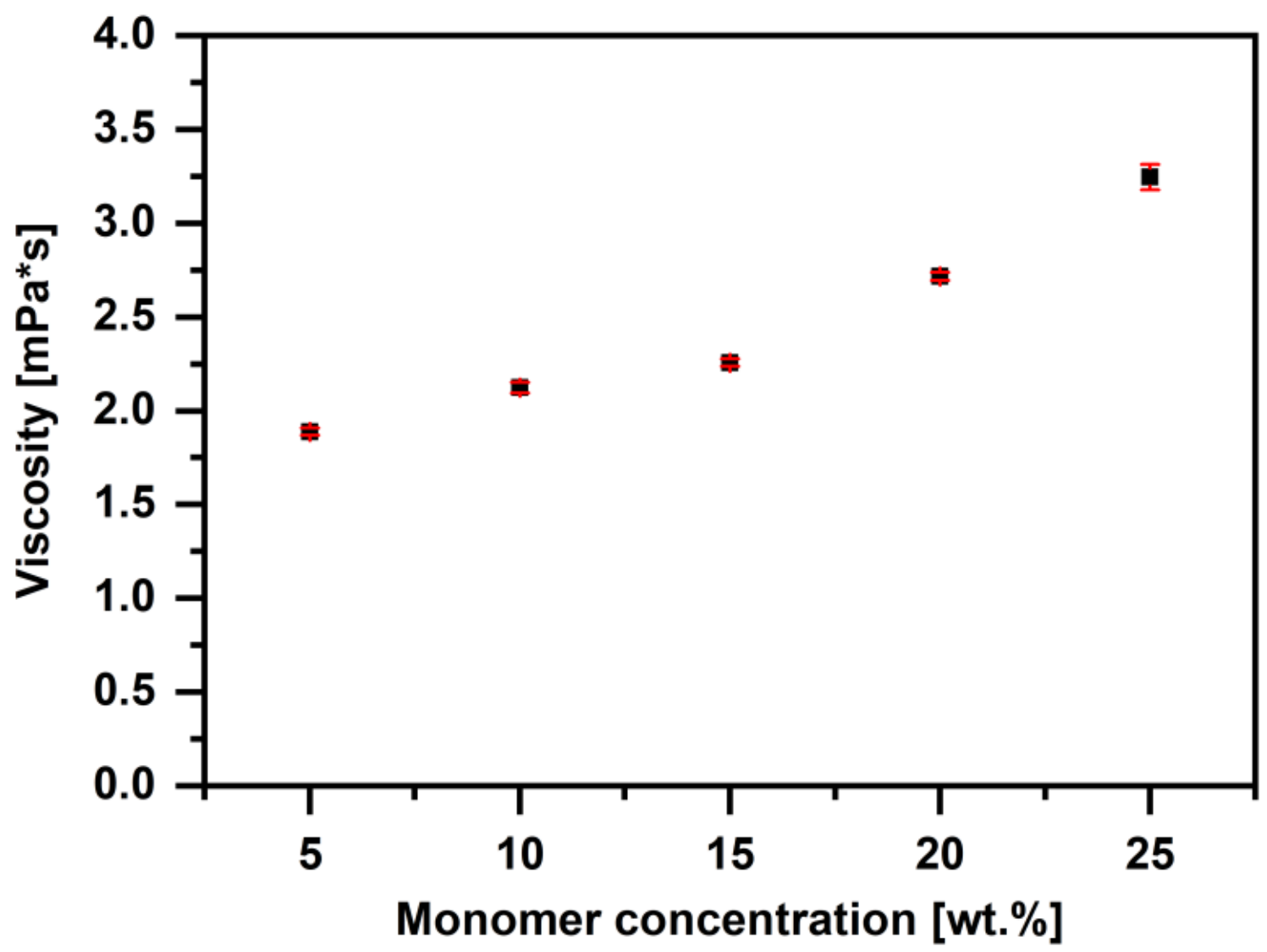

Figure S5: Dynamic viscosity of NAG monomer solutions at varying concentrations (5–25 wt.%) at 25 °C. Measurements were conducted with a rheometer in continuous rotation mode over a range of shear rates from 10^{-2} to 17,900 s^{-1}. The solutions exhibited Newtonian behavior, with viscosity stabilizing over the shear rates from 10^2 to 10^4 s^{-1}. The average viscosity within this stabilized region was calculated for each run, and the mean and standard deviation were determined across independent measurements. Data are presented as mean ± standard deviation (n = 3).

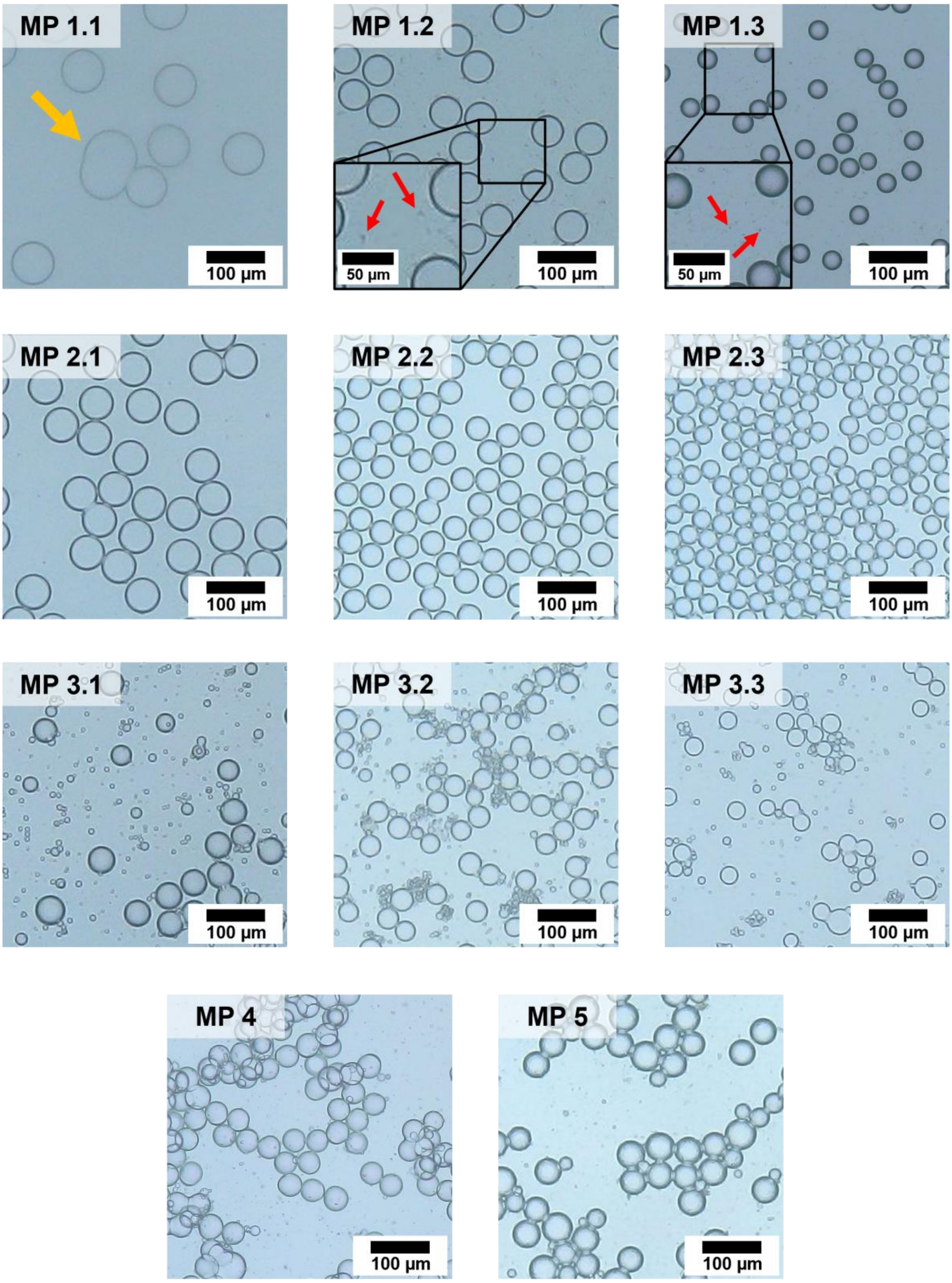

Figure S6: Optical microscopy images of microparticles (MP 1-MP 5) dispersed in oil, produced under different monomer phase compositions and flow rates. The orange arrow in MP 1.1 indicates an irregularly shaped particle. Insets in MP 1.2 and MP 1.3 provide a zoom-in to highlight the presence of debris, with larger agglomerates marked by red arrows.

Supporting References

1. Gao, F.; Zhang, Y.; Li, Y.; Xu, B.; Cao, Z.; Liu, W., *ACS Applied Materials & Interfaces* **2016,** *8* (14), 8956-66. .
2. Phadke, A.; Zhang, C.; Arman, B.; Hsu, C. C.; Mashelkar, R. A.; Lele, A. K.; Tauber, M. J.; Arya, G.; Varghese, S., *Proceedings of the National Academy of Sciences* **2012,** *109* (12), 4383-4388.
3. Shah, R. K.; Shum, H. C.; Rowat, A. C.; Lee, D.; Agresti, J. J.; Utada, A. S.; Chu, L.-Y.; Kim, J.-W.; Fernandez-Nieves, A.; Martinez, C. J.; Weitz, D. A., *Materials Today* **2008,** *11* (4), 18-27.
4. Saalwächter, K.; Ziegler, P.; Spyckerelle, O.; Haidar, B.; Vidal, A.; Sommer, J. U., *Journal of Chemical Physics* **2003,** *119*, 3468-3482.
5. Lange, F.; Schwenke, K.; Kurakazu, M.; Akagi, Y.; Chung, U.-i.; Lang, M.; Sommer, J.-U.; Sakai, T.; Saalwächter, K., *Macromolecules* **2011,** *44* (24), 9666-9674.
6. Lamsal, B.; Löser, L.; Saalwächter, K., *Macromolecular Chemistry and Physics* **2026,** *227* (7), e70240.
7. Newville, M.; Otten, R.; Nelson, A.; Stensitzki, T.; Ingargiola, A.; Allan, D.; Fox, A.; Carter, F.; Rawlik, M. *LMFIT: Non-Linear Least-Squares Minimization and Curve-Fitting for Python*, Zenodo: **2025**.
